# DNA sequence-dependent activity and base flipping mechanisms of DNMT1 regulate genome-wide DNA methylation

**DOI:** 10.1038/s41467-020-17531-8

**Published:** 2020-07-24

**Authors:** Sabrina Adam, Hiwot Anteneh, Maximilian Hornisch, Vincent Wagner, Jiuwei Lu, Nicole E. Radde, Pavel Bashtrykov, Jikui Song, Albert Jeltsch

**Affiliations:** 1 Department of Biochemistry, Institute of Biochemistry and Technical Biochemistry, University of Stuttgart, Allmandring 31, 70569 Stuttgart, Germany; 2 Department of Biochemistry, University of California, Riverside, CA 92521 USA; 3 Institute for Systems Theory and Automatic Control, University of Stuttgart, Pfaffenwaldring 9, 70569 Stuttgart, Germany

**Keywords:** Enzyme mechanisms, DNA methylation, X-ray crystallography

## Abstract

DNA methylation maintenance by DNMT1 is an essential process in mammals but molecular mechanisms connecting DNA methylation patterns and enzyme activity remain elusive. Here, we systematically analyzed the specificity of DNMT1, revealing a pronounced influence of the DNA sequences flanking the target CpG site on DNMT1 activity. We determined DNMT1 structures in complex with preferred DNA substrates revealing that DNMT1 employs flanking sequence-dependent base flipping mechanisms, with large structural rearrangements of the DNA correlating with low catalytic activity. Moreover, flanking sequences influence the conformational dynamics of the active site and cofactor binding pocket. Importantly, we show that the flanking sequence preferences of DNMT1 highly correlate with genomic methylation in human and mouse cells, and 5-azacytidine triggered DNA demethylation is more pronounced at CpG sites with flanks disfavored by DNMT1. Overall, our findings uncover the intricate interplay between CpG-flanking sequence, DNMT1-mediated base flipping and the dynamic landscape of DNA methylation.

## Abstract (web summary)

DNA methylation is one of the major epigenetic mechanisms that critically influence gene expression, genomic stability and cell differentiation. Here, the authors study DNMT1 in complex with DNA substrates and systematically analyze the mechanism and specificity of DNMT1.

## Introduction

DNA methylation plays essential biological roles in gene regulation and chromatin biology^1–3^. In the human genome, DNA methylation occurs at about 70–80% of all CpG sites^4^. It is established by the de novo DNA methyltransferases DNMT3A and DNMT3B^5,6^, and maintained by DNA methyltransferase 1 (DNMT1)^7,8^ in a replication-coupled manner^9,10^. Previous studies have demonstrated that DNMT1-mediated maintenance DNA methylation is ensured by a multilayered regulation^11^. Notably, Ubiquitin-like containing PHD and RING Finger domains 1 (UHRF1) plays an essential role in genomic targeting of DNMT1^12,13^. In addition, the activity of DNMT1 in maintenance DNA methylation is supported by its substrate preference for hemimethylated CpG sites, as well as a high level of enzymatic processivity^14–18^. Due to lack of systematic structural and kinetic characterizations, the mechanistic principles underlying the target specificity of DNMT1 and its connection with genomic methylation patterns remain elusive.

The recognition site of mammalian DNMTs, a CpG dinucleotide, is short when compared with recognition sites of transcription factors or enzymes of restriction modification systems. However, increasing evidence has indicated that the activity of DNMTs can be strongly influenced by sequences outside the core CpG site, called flanking sequence here. For instance, the DNMT3 enzymes have been reported for their pronounced flanking sequence preferences^19–25^. Recently, it has even been demonstrated that alteration of the flanking sequence preferences of DNMT3A provides a key mechanistic basis for cancer promoting effects of the somatic DNMT3A R882H mutation, which is frequently observed in acute myeloid leukemia (AML)^23,24^. In contrast to the well-documented flanking sequence effects on DNMT3A and DNMT3B, there is no systematic analysis of potential flanking sequence preferences of DNMT1.

In order to elucidate the biochemical and enzymatic principles of DNMT1 activity, we analyzed DNMT1-mediated methylation of long hemimethylated DNA molecules by bisulfite conversion coupled to Next Generation Sequencing (NGS). By combining in-depth sequencing analysis, biochemical characterization, and quantitative modeling based on stochastic chemical reaction kinetics, we identified details of the kinetic mechanism of DNMT1. Importantly, we observed a strong flanking sequence effect on DNMT1, which was further confirmed by methylation of a library of substrates, containing one hemimethylated CpG site in a randomized sequence context. In addition, we determined the crystal structures of DNMT1 in complex with DNA substrates with different sequence contexts, which provide an explanation for the flanking sequence-based preferences of DNMT1 at atomic level and revealed flanking context dependent base flipping mechanisms of DNMT1. Moreover, we show that the flanking sequence profiles of DNMT1 are highly correlated with genomic methylation patterns in human and mouse cells, suggesting that flanking sequence preferences of DNMT1 shape genome-wide DNA methylation patterns.

## Results

### DNA methylation kinetics using a hemimethylated substrate

Kinetic studies have revealed that DNMT1 has a processive reaction mechanism, in which it methylates many hemimethylated CpG sites without dissociating from the DNA^16,26,27^. The biochemical observation that processive methylation occurs on only one DNA strand indicates that DNMT1 moves on the DNA by linear diffusion during the processive reaction^16^. However, details of this mechanism, in particular the level of processivity, have remained unclear. Moreover, highly processive enzymes, as exemplified by the polymerase processivity factor PCNA or DNA helicases, often undergo conformational transitions during the reaction cycle, in which they adopt a closed conformation for processive reaction and movement on the DNA, but an open conformation for DNA binding or release^28,29^. However, such conformational changes have not yet been identified for DNMT1.

To improve our understanding of the kinetic properties of DNMT1, deep enzymology experiments were conducted. With this term, we refer to enzymatic assays on DNA with a very detailed readout of millions of single molecule product methylation patterns by deep sequencing. As a methylation substrate, a 349 bp long hemimethylated DNA derived from the CpG island upstream of the human ZNF280B gene was synthesized using the approach described in Supplementary Fig. 1a. In brief, the unmethylated substrate was PCR amplified from a plasmid containing the cloned target sequence using a phosphorylated primer for the upper DNA strand and a lower strand primer containing phosphorothioate linkages. Next, the purified PCR product was methylated with M.SssI at all CpG sites in both DNA strands (fully methylated). Afterwards, the fully methylated substrate was digested with lambda 5′→3′ exonuclease, which preferentially cleaves the upper DNA strand containing phosphorylated 5′-ends, but not the lower strand protected by the phosphorothioate linkages, generating a single-stranded methylated DNA. Finally, a hemimethylated double-stranded substrate was generated by primer extension using the methylated single-stranded DNA as template. The final substrate contained 44 hemimethylated CpG sites, which are methylated in the lower DNA strand (Supplementary Fig. 2).

**Fig. 1:**
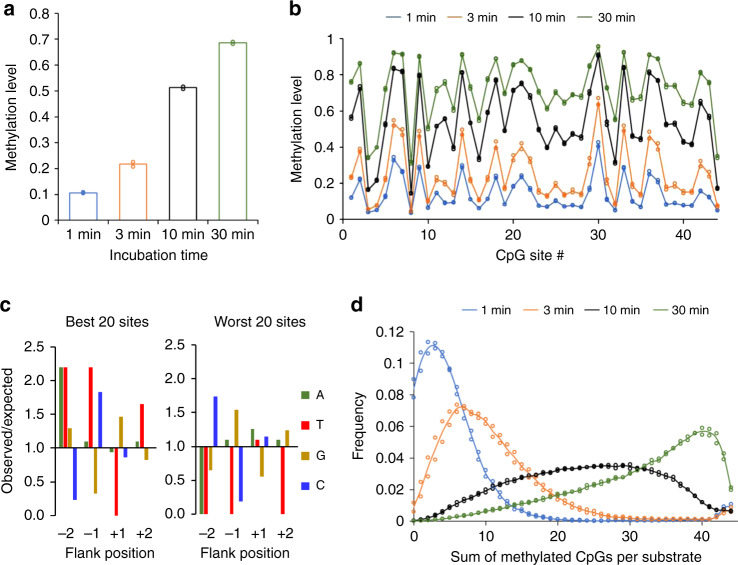
Deep enzymology methylation data of the hemimethylated 349 bp substrate. **a** Time course of the overall methylation levels. **b** Time dependence of the methylation profile of the 44 CpG sites. **c** Enrichment and depletion of individual bases at different flanking positions in the most preferred and most disfavored CpG sites in (**b**). **d** Single molecule analysis showing the fraction of DNA molecules with different number of methylated CpG sites. The bars in (**a**), (**b**), and (**d**) display the average of two independent experiments. Source data are provided as a Source Data file.

The hemimethylated DNA was methylated by murine DNMT1 (0.19 µM) for different incubation times (1, 3, 10, and 30 min) in two independent reactions. After bisulfite conversion of all samples, libraries for NGS were prepared by adding barcodes and indices. Bisulfite treated, unmethylated DNA was included as control showing bisulfite conversion rates of >99.5% (Supplementary Table 1). Bisulfite analysis of the methylated strand confirmed high methylation levels (>99%). As an additional control, bisulfite analysis of the unmethylated strand of the hemimethylated substrate was conducted revealing methylation levels of 1–2%. These methylation events were all clustered on single DNA molecules, indicating that they originate from small amounts of carryover of the original fully methylated template DNA. As illustrated in Fig. 1a, b, d, the two experimental repeats of DNMT1 methylated samples revealed very similar results. Moreover, a strong correlation was observed between global methylation levels and incubation times (Fig. 1a). Strikingly, the different CpG sites showed different methylation rates, some of them showing methylation levels >40% already after 1 min while others still showed <15% methylation after 10 min of methylation, accounting for a ~30-fold difference in methylation rates (Fig. 1b). An analysis of the enrichment and depletion of individual bases at the flanking positions of the preferred and disfavored sites revealed strong and opposite effects (Fig. 1c) suggesting that the different methylation rates were mostly caused by the different flanking sequences.

### Quantitative modeling of DNA methylation by DNMT1

**Fig. 2:**
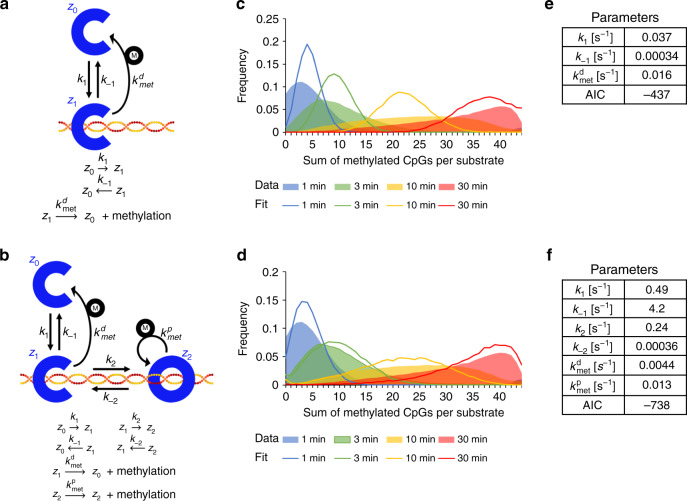
Fitting of the experimental methylation data reveals a processive methylation mechanism. Schematic drawing of the kinetics models of the DNA methylation mechanism of DNMT1 used for fitting of experimental data. We used a purely distributive model (Model 1, **a**) or a mixed model including distributive and processive methylation (Model 2, **b**). The corresponding reactions are indicated below the schemes. Comparison of the experimental data (colored areas) and best fit (colored lines) for the distributive (**c**) and mixed (**d**) models. Model parameters after fitting for the distributive (**e**) and mixed (**f**) models. AIC, Akaike Information Criterion.

We further analyzed the single molecule methylation data and extracted the cumulative methylation levels of individual substrate molecules (repeat 1 and repeat 2 in Supplementary Table 1 and Fig. 1d). Different kinetic models based on stochastic chemical reaction kinetics were built and calibrated to the frequency distribution of the number of methyl groups per DNA molecule using Gillespie’s algorithm to simulate sample paths of the respective Chemical Master Equation^30,31^. We applied a simple model (Model 1) in which DNMT1 methylates DNA in an obligate distributive mechanism (Fig. 2a) and a second model (Model 2) in which DNA bound DNMT1 can reversibly switch from an open to a closed conformation (Fig. 2b). In the closed conformation, DNMT1 does not dissociate from the DNA and can methylate different CpG sites in a processive reaction. Both models treat DNA methylation with site resolution and consider the flanking sequence preferences of DNMT1 (for details refer to “Materials and methods” and Supplementary Note 1). Model fits are depicted in Fig. 2c for Model 1 and Fig. 2d for Model 2, which clearly illustrate that Model 1 is not able to capture the experimental methylation dynamics qualitatively. In particular, the variance of the distribution predicted by Model 1 was significantly smaller than the empirical variance of the experimental frequency distributions. In contrast, Model 2 provides a visually good fit, which is a striking result given the relatively small number of parameters used in this model. In Model 2, the wide variance of the experimental data is captured due to two effects. Processive methylation leads to a consecutive methylation of several sites, which results in an over-proportional generation of DNA molecules with high methylation. In addition, processive methylation sequesters DNMT1 away from yet unbound DNA molecules, thus increasing the fraction of DNA molecules with zero or low methylation. Since Model 1 is a submodel of Model 2, we also compared both models by using Akaike’s Information Criterion (AIC)^32^, which penalizes more complex models, resulting in a highly significant preference for Model 2 (Supplementary Note 1). This analysis supports our conclusion that DNA methylation of DNMT1 can only be described by a processive reaction mechanism.

Estimated model parameters are listed in Fig. 2e for Model 1 and Fig. 2f for Model 2. The estimated parameters of Model 2 provide insights into DNMT1-mediated methylation mechanisms and can be interpreted as follows: first, *k*_1_ and *k*_−1_ are the dominant reaction rate constants, meaning that the most rapid reactions of DNMT1 are binding to and dissociation from the DNA in the open conformation. Second, comparison of the processive and distributive DNA methylation rates indicates that processive methylation is about three times faster. Third, comparison of *k*_−1_, $\it {\mathit{k}}_{{\mathrm{met}}}^{\mathrm{d}}$ and *k*_2_ indicates that, in the initial phase of the reaction, 95% of the DNMT1 molecules will dissociate from the DNA after DNA binding without methylation, 5% will change into the closed conformation, and <0.1% will methylate the DNA in a distributive reaction. Similarly, comparison of ${\mathit{k}}_{{\mathrm{met}}}^{\mathrm{p}}$ and *k*_−2_ indicates that DNMT1, once in the closed conformation, has a propensity of 97% for processive methylation in the initial reaction phase. Overall, our modeling study clearly shows that DNMT1 methylates DNA predominantly in a processive manner.

### Flanking sequence preference analysis of DNMT1

To investigate flanking sequence preferences of DNMT1 in more detail, we resorted to our recently developed deep enzymology workflow allowing us to study the methylation of CpG sites in a randomized flanking sequence context^24,25^ (Supplementary Fig. 1b). We first generated a pool of double-stranded DNA substrates, which contained one hemimethylated CpG site flanked by 10 random base pairs on either side (Supplementary Fig. 3). Then, the pool of substrates was methylated with DNMT1 and subjected to hairpin ligation and bisulfite conversion, followed by two consecutive PCR reactions with only few cycles for library generation, which also added barcodes and indices^33^. Subsequently, different product libraries were mixed and sequenced by NGS (Supplementary Table 2). Controls using unmethylated and hemimethylated DNA revealed high efficiencies of bisulfite conversion and high preferences of DNMT1 for hemimethylated CpG sites (Supplementary Table 2).

**Fig. 3:**
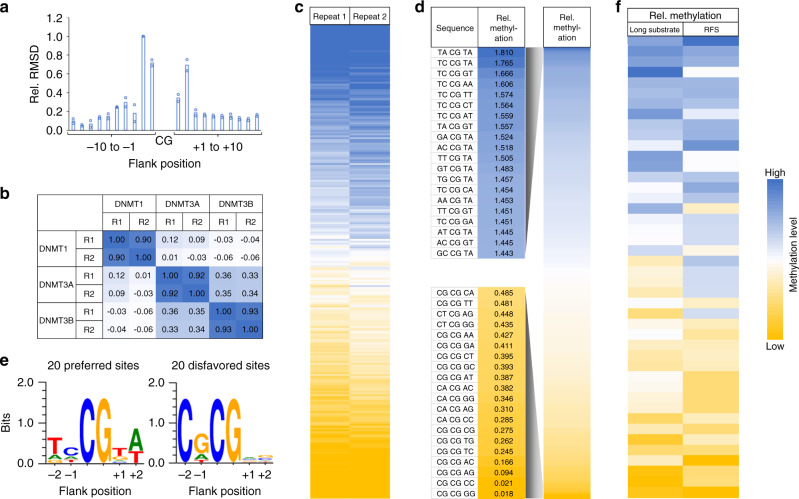
Deep enzymology analysis of the flanking sequence preferences of DNMT1. **a** Relative base preferences of DNMT1 at the −10 to +10 flanking positions surrounding the CpG site. The numbers refer to the root mean squared deviations (RMSD) of the observed/expected base composition at each site among the methylated sequence reads, normalized to the highest effect observed at position −2. The bars show averages of two independent experiments. **b** Methylation levels were obtained from two experimental repeats (R1 and R2), averaged for all NNCGNN flanking sites and the Pearson correlation coefficients (*r* values) of the pairwise comparison of the data sets were determined. DNMT3A and DNMT3B data were taken from our previous study for comparison^25^. **c** The methylation levels of NNCGNN sites of both repeats of the DNMT1 methylation reaction displayed as heatmap to show the strong correlation of both data sets (see also Supplementary Fig. 3b). **d** Heatmap of the methylation levels of NNCGNN flanking sites ordered by decreasing DNMT1 activity. The outlines show the 20 most favored or disfavored flanking sites. **e** Sequence logos of the 20 flanking sites most favored or disfavored by DNMT1. **f** Comparison of the NNCGNN methylation levels determined for the 44 CpG sites on the long substrate (long substrate) with the methylation levels determined for the corresponding NNCGNN sites in random flank library methylation experiment (random flank substrate—RFS). The image shows the methylation levels of individual NNCGNN sites as heatmap. The *p* value of the correlation is 1.25 × 10^−4^. The *p* value is based on the Pearson correlation coefficient (*r*-factor) and the *Z*-statistics of the distribution of *r*-factors determined after randomization of one of the data sets. See also Supplementary Fig. 4b for a box plot comparison. Source data are provided as a Source Data file.

Two independent methylation reactions (repeat 1 and repeat 2) were performed with DNMT1 and sequenced at great depth to investigate the details of the flanking sequence preferences of DNMT1 (Supplementary Table 2). To determine the overall influence of each flanking position on enzymatic activity, observed and expected frequencies of each nucleotide were determined at each flanking site in the methylated products. This analysis showed that the activity of DNMT1 was strongly influenced by the −2 to +2 flanking sequence of the CpG site (Fig. 3a). Therefore, we focused on further analyzing the effect of the ±2 bp (N2 = NNCGNN) flanking positions on the activity of DNMT1 and determined the average methylation for all 256 N2 flanks. In our previous work, two sets of methylation reactions with random flank substrates were carried out with the catalytic domains of DNMT3A and DNMT3B using the same substrate library and experimental approach^25^, which were used for comparison here. Correlation analysis of the DNMT1 methylation profiles with the previously obtained data for DNMT3A and DNMT3B showed that the repetitions of experiments with the same enzyme were always highly correlated (Fig. 3b). The close correlation of the two DNMT1 experimental repeats is also visible in heatmaps of the methylation levels of NNCGNN sites (Fig. 3c). In contrast, the average methylation levels of CpG sites in different flanking contexts were only weakly correlated between DNMT1 and DNMT3A or DNMT3B (Fig. 3b). As shown in the Supplementary Table 2, DNMT1 repeat 2 reached higher overall methylation than repeat 1, which is in agreement with the higher enzyme concentration used in this reaction. However, based on the correlation analyses shown in Fig. 3b, c, both data sets were normalized for their average methylation and merged for further analyses. The averaged and normalized flanking sequence preferences revealed very strong (almost 100-fold) differences in the relative preferences of DNMT1 for NNCGNN flanks (Fig. 3d). Consistent results were obtained in a Weblogo analysis (http://weblogo.threeplusone.com/) prepared using the 20 N2 flanks most preferred and most disfavored by DNMT1 (Fig. 3e).

### Validation of the NGS derived sequence preference profiles

To validate the flanking sequence effects observed in the methylation of the random flank substrate library, two pairs of substrates were designed on the basis of the DNMT1 preference profiles always combining one substrate that was expected to be preferred and one expected to be disfavored. Using these substrates and a reference substrate taken from our previous work^15,18,34^ that was mildly disfavored (NNCGNN preference rank 170 of 256, where rank 1 represents the highest preference), the rates of methylation of the hemimethylated CpG site were determined by measuring the transfer of methyl groups from radioactively labeled S-Adenosyl-L-Methionine (AdoMet) to the DNA. As shown in Table 1 and Supplementary Fig. 4a, the experimental methylation rates were in very good agreement with the flanking sequence data. The favorable substrates were methylated about 4-fold faster than the reference substrate and the unfavorable substrates had methylation rates about 3-fold slower, amounting to roughly 12-fold differences in methylation rates. These effects were smaller than those observed after methylation of the pool of random flank substrates, likely due to the different experimental conditions between the two systems. In the radioactive kinetic studies with the model substrates, each substrate was investigated separately. In contrast, in the methylation of the pool of random flank substrates, all different substrates were present at the same time and the enzyme made its choice among them, which is expected to enhance the differences.

**Table 1:** Validation of the NGS profiles.

Sequence	Rank in NNCGNN profile	Relative methylation (mean ± SD)
GTA**CG**TAA	1	3.77 ± 0.50
TTC**CG**TAA	2	4.32 ± 0.43
TCC**CG**GAA	170	1.0
TCG**CG**AGC	254	0.36 ± 0.03
TCG**CG**AGA	254	0.33 ± 0.04

NGS derived flanking sequence preferences were validated by radioactive DNA methylation kinetics with exemplary substrates. All sequences were embedded in a constant TTGCACTCTCCNNNCGNNNGTCCCAGCTTC flanking sequence context outside of the ±3 flanking region and annealed with methylated complementary strand. Substrate preferences of DNMT1 in the deep enzymology experiments are indicated by the rank of the substrate in the NNCGNN profile, where 1 represents the highest preferences and 256 the lowest. Methylation rates are presented as averages and standard deviations of three independent experiments. Representative primary data are show in Supplementary Fig. 4a. The central CpG site is printed in bold letters and the N2 flanks are underlined.

The flanking sequence preferences derived from the random flank analysis agree well with the flanking sequence preference profiles derived from the 44-site substrate methylation data described above. Specifically, in the 44-site data set, C at the −2 and G at the −1 site were disfavored while G at the +1 site and T (plus weakly A) at the +2 site were favored by DNMT1 (Fig. 1c). All these effects were recapitulated in the detailed analysis based on the deep enzymology data (Fig. 3e). Some differences in the enrichment or depletion of individual bases in both data sets can be explained by the low representation of these bases at the corresponding positions in the 44 CpG sites which does not provide a reliable statistical basis (total occurrence A at −2: 2, T at −1: 2, T at +1: 2, A and T at +2: 4). The agreement between both data sets is also illustrated by comparison of the relative methylation rates observed at all sites on the 44-site substrate with the rates observed with the random flank substrate for the corresponding N2 flank, which shows a high congruence (Fig. 3f and Supplementary Fig. 4b). To determine the statistical significance of this correlation, the analysis was repeated with randomly shuffled random flank data. Based on the distribution of the Pearson correlation coefficients (*r* values) in these random correlations, a *p* value of 1.25 × 10^−4^ was determined, indicating that the observed correlation of both data sets is highly significant.

### Structure of DNMT1 bound to different DNA substrates

Next we asked, if the CpG-flanking sequences of DNA substrates impact the protein-DNA interaction of DNMT1. In this regard, we previously solved the crystal structure of a complex between a C-terminal fragment of mouse DNMT1 (residues 731–1602, mDNMT1_731–1602_) and a hemimethylated DNA. Control experiments revealed the same flanking sequence preferences of mDNMT1_731–1602_ as those of full-length mDNMT1 (Supplementary Fig. 4c, d). The DNA used for crystallization contained a central CpG site where the target cytosine was replaced by 5-fluorocytosine (5fC) (PDB 4DA4)^17^. The presence of 5fC in the CpG site permits the formation of a covalent, productive complex between DNMT1 and DNA, which was used for structural characterization^35^. This DNA contained a GGCGGC sequence (GCG complex), which represents a relatively disfavored NNCGNN flank (rank 177 of 256, where rank 1 represents the highest preference). We therefore applied the same approach to obtain the covalent complexes of DNMT1 with two relatively preferable substrates, containing TCCGTA (rank 2, CCG complex) and TACGGA (rank 32, ACG complex) sequences, respectively (Table 2). We generated the crystals for both complexes under crystallization conditions similar to that used for the GCG complex^17^. The crystal structures of the CCG and ACG DNA complexes of DNMT1, each bound to S-Adenosyl-L-homocysteine (AdoHcy), an analog of AdoMet, were solved at 3.0 Å and 3.1 Å resolution, respectively (Supplementary Table 4). Additional attempts to obtain a sufficient amount of the complex between DNMT1 and a highly disfavored CGCGAG sequence (rank 254) (Table 2) for crystallization failed, presumably due to the low affinity of DNMT1 for this sequence and/or low crosslinking yield with 5fC within this highly disfavored DNA sequence.

**Table 2:** Oligonucleotide sequences used for DNMT1 structural analyses.

Sequence	Rank in NNCGNN profile	PDB number	Reference
TTC**CG**TAA	2	6W8W	This study
TTA**CG**GAA	32	6W8V	This study
AGG**CG**GCC	177	4DA4	Ref. ^17^
TCG**CG**AGA	254	–	This study

Substrate preferences of DNMT1 determined in the deep enzymology experiments are indicated by the rank of the substrate in the NNCGNN profile, where 1 represents the highest preference and 256 the lowest. The central CpG site is printed in bold letters and the N2 flanks are underlined.

**Fig. 4:**
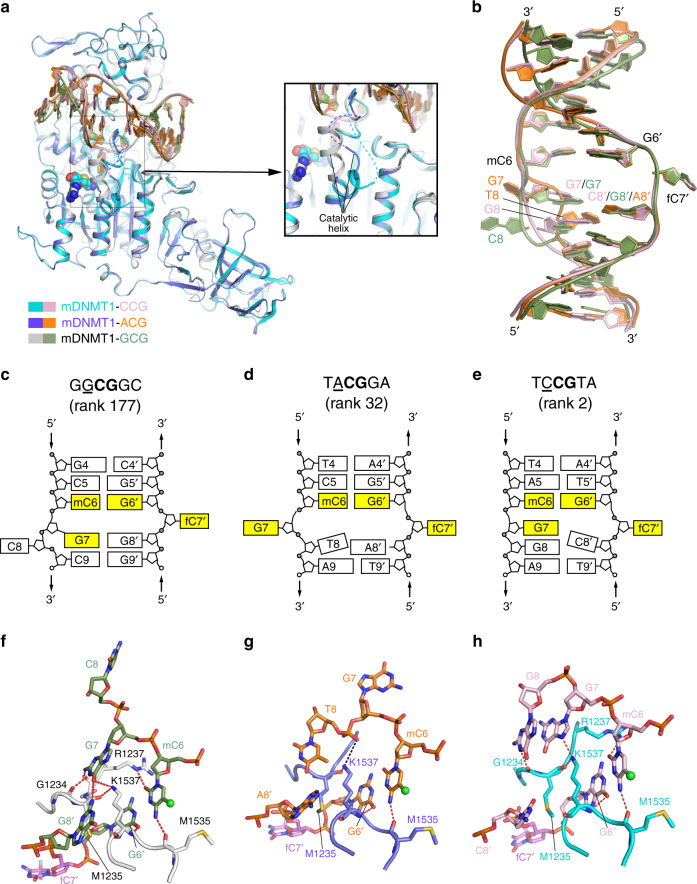
Structural analysis of the murine DNMT_731–1602_-DNA complexes. **a** Structural overlay of the CCG and ACG complexes (this study) and the GCG complex^17^ (PDB 4DA4) with the catalytic helix highlighted in expanded view. The disordered regions N-terminal to the catalytic helix are shown as dashed lines in the respective CCG and ACG complexes. The AdoHcy molecule is shown in sphere representation. **b** Structural overlay of the CCG DNA, ACG DNA and GCG DNA in DNMT1-bound form. The color scheme is the same as in (**a**). Schematic views of the DNMT1- bound DNAs in the GCG complex (**c**), adopted from a previous report^17^, and ACG (**d**) and CCG (**e**) complexes. Close-up comparison of the protein-DNA interactions at the CpG sites between the GCG (**f**), ACG (**g**) and CCG (**h**) complexes. The 5-methyl group of the mC6 is shown as green sphere. The hydrogen-bonding and electrostatic interactions are shown as dashed lines in red and black, respectively. Note that in the ACG complex, the side chain of R1237 is non-traceable due to missing electron density.

Structural comparison of the CCG and ACG complexes of DNMT1 with the previously determined GCG complex reveals high overall structural similarity, with RMSD values of 0.40 and 0.34 Å over 784 and 788 aligned Cα atoms, respectively (Fig. 4a). In all three complexes, the target 5fC (fC7′) is flipped out of the DNA duplex and inserted into the active site of DNMT1, anchored through the covalent linkage with the catalytic cysteine C1229 and hydrogen-bonding interactions with other catalytic residues (Supplementary Fig. 5). Nevertheless, significant structural differences among the three complexes were observed for the bound DNA molecules, which undergo different conformational reorganization following base flipping of fC7′ (Fig. 4b). Moreover, the helix C-terminal to the catalytic cysteine C1229 (catalytic helix, residues 1241–1262 of DNMT1) shifts from a mainly straight conformation in the GCG complex to a mainly kinked conformation in the CCG and ACG complexes (Fig. 4a).

As observed previously, the intermolecular interaction between DNMT1 and the DNA is mainly mediated by two loops from the target recognition domain of DNMT1 (TRD loop I: residues 1501–1516; TRD loop II: residues 1530–1538) and the catalytic loop (residues 1226–1240), engaging the DNA from the major groove and minor groove sides, respectively (Supplementary Fig. 6). Overall, the structures of the CCG and ACG complexes of DNMT1, despite with moderate resolution that precludes detailed analysis of water-mediated interactions, reveal protein-DNA interactions similar to those of the GCG complex (Fig. 4f–h and Supplementary Fig. 6a–c), which in all three complexes are associated with disruption of the Watson-Crick base pairs at the target fC7′ (Fig. 4b, f–h). However, the two new structures revealed distinct patterns of base re-positioning accompanying flipping of the target base. In the unfavorable GCG complex (Fig. 4f), the orphan guanine that normally pairs with the target cytosine fC7′ is held inside the DNA helix by a hydrogen-bonding interaction with G8′ of the target strand, as well as hydrogen-bonding interactions with G1234 and M1235 on the catalytic loop and K1537 from the TRD loop II. The formation of the noncanonical G7–G8′ base pair in turn leads to flipping out of the cytosine at the −1 site on the nontarget strand (C8). In contrast, in the ACG complex (Fig. 4g), base flipping of target fC7′ is accompanied by flipping out of the orphan G7 on the nontarget strand, rather than the −1 base (T8), presumably due to the loss of base pairing between G7 and the −1 base on the target strand. Interestingly, in the CCG complex (Fig. 4h), the orphan guanine G7, while being held inside the DNA helix via a hydrogen-bonding interaction with K1537, also did not pair with the −1 base on the target strand (C8′); instead, it stacks against the −1 base on the nontarget strand (G8), resulting in no base eviction from the nontarget strand. Together, these structures highlight the conformational dynamics of the DNA following base flipping of target fC7′ and its dependence on the flanking sequence context at the minus side of the CpG site.

**Fig. 5:**
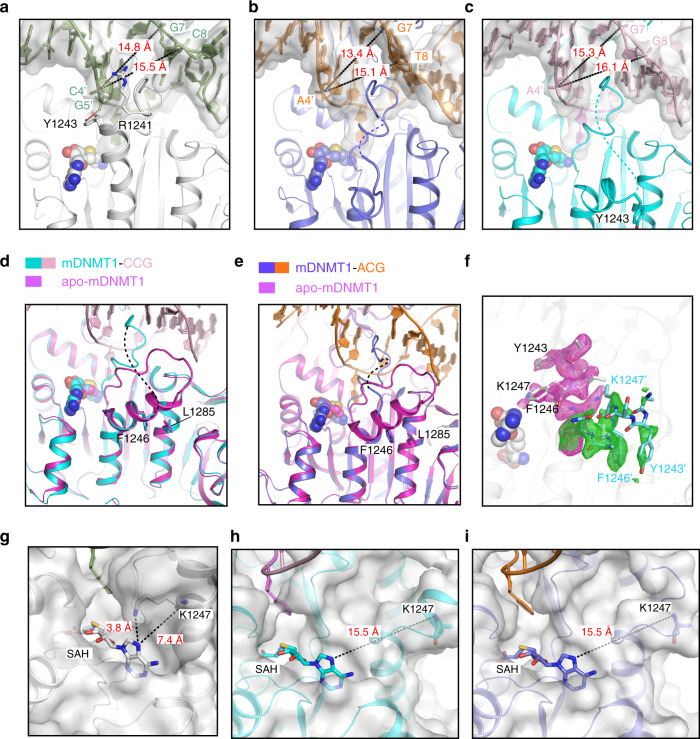
Conformational transition of the catalytic helix of murine DNMT1. Close-up views of the conformation of the catalytic helix in the GCG (PDB 4DA4) (**a**), ACG (**b**), and CCG (**c**) complexes. The side chains of the DNA-interacting residues R1241 and Y1243 are shown in stick representation in the GCG complex (**a**). These two residues are either completely (in the ACG complex) or partially (CCG complex) non-traceable in the other two complexes. The hydrogen-bonding interaction is shown as dashed line in red. The minor groove width at the +2 flank sites (C4′/A4′) is indicated by dashed lines in black. The disordered segments in (**b**) and (**c**) are shown by dashed lines in slate and cyan, respectively. Close-up view of the catalytic helix overlaid between a DNMT1 structure with no DNA bound to the catalytic domain (PDB 3PT9) and the CCG complex (**d**) or the ACG complex (**e**). Fo-Fc omit map of residues 1242–1249 of mDNMT1_731–1602_ in the GCG DNA complex (PDB 4DA4). The straight conformation and associated map (2.0 σ level) are colored in silver and magenta, respectively. The kinked conformation and associated map (1.9 σ level) are colored in aquamarine and green, respectively. Enlargement of the AdoMet-binding pockets in the GCG (**g**), CCG (**h**) and ACG (**i**) complexes. The distances between the N7 atom of AdoHcy (SAH) and the side chain or the backbone of residue K1247 are indicated by dashed lines. Note that the side chain of K1247 is non-traceable in (**h**–**i**) due to missing electron density.

Unlike the GCG complex, in which the catalytic helix is mainly in a straight conformation^17^ (Figs. 4a and 5a), the catalytic helix in the CCG and ACG complexes dominantly adopt a kinked conformation (Figs. 4a and 5b, c), reminiscent of what has been observed in the structure of DNMT1 with no DNA bound to the catalytic domain^36^ (Fig. 5d, e). Accompanying this conformational shift, no traceable electron density was observed for the segment immediately upstream of the catalytic helix in the CCG and ACG complexes (residues 1239–1241 in the ACG complex and residues 1239–1243 in the CCG complex) suggestive of structural disorder of this region (Fig. 5b, c). Such a conformational difference of the catalytic helix between different DNMT1-DNA complexes suggests that it may undergo dynamic exchange between the straight and kinked conformations during catalysis. Indeed, detailed analysis of the electron density of the three complexes revealed that the GCG complex also contains a minor population of the kinked conformation (Fig. 5f), while the ACG and CCG complexes both contain a minor population of the straight conformation (Supplementary Fig. 7), in addition to their respective major population of the kinked conformations. Notably, in the GCG complex, the cofactor binding pocket is partly sealed by K1247 from the catalytic helix (Fig. 5g), whereas it becomes more solvent accessible in the CCG and ACG complexes due to the fact that K1247 moves away as the catalytic helix transitions into the kinked conformation (Fig. 5h, i). This change in solvent accessibility of the AdoMet-binding pocket presumably provides a regulatory mechanism for cofactor association and dissociation of DNMT1 during catalysis, supporting a previous report that the kinked-to-straight conformational transition is required for DNMT1-mediated DNA methylation^37^.

The conformational transition between these two alternative states of the catalytic helix is likely driven by the interplay between the intermolecular and intramolecular interactions of DNMT1. In particular, the straight conformation is stabilized by the contacts involving R1241 and Y1243 of DNMT1 and the DNA backbone phosphate of C4′ and G5′ in the +1 and +2 flanking positions (Fig. 5a), while the kinked conformation is stabilized by the packing interaction between F1246 and L1285 of DNMT1 (Fig. 5d, e). In this regard, a subtle conformational change in the bound DNA (e.g., variation in the minor groove width (MGW)) may influence the equilibrium between the two alternative conformations (Fig. 5a–c), which may explain the pronounced effects of residues at the plus-side flank of the CpG site on the catalytic activity of DNMT1. To investigate the potential influence of the MGW on the flanking sequence preferences of DNMT1, the DNA shape prediction server has been used (http://rohslab.cmb.usc.edu/DNAshape/)^38^. It predicts the MGW for base pairs considering the two neighboring base pairs in both directions. At each position, the average MGW was determined for the methylated molecules and for all molecules, and the difference of these numbers was calculated (Supplementary Fig. 8). The results show a negative peak at the CpG site, but strong and highly significant positive peaks were observed for the +1 to +3 flank, indicating that methylated DNA molecules had an increased MGW at the plus-side flank.

### Correlation of flanking preferences with genomic methylation

**Fig. 6:**
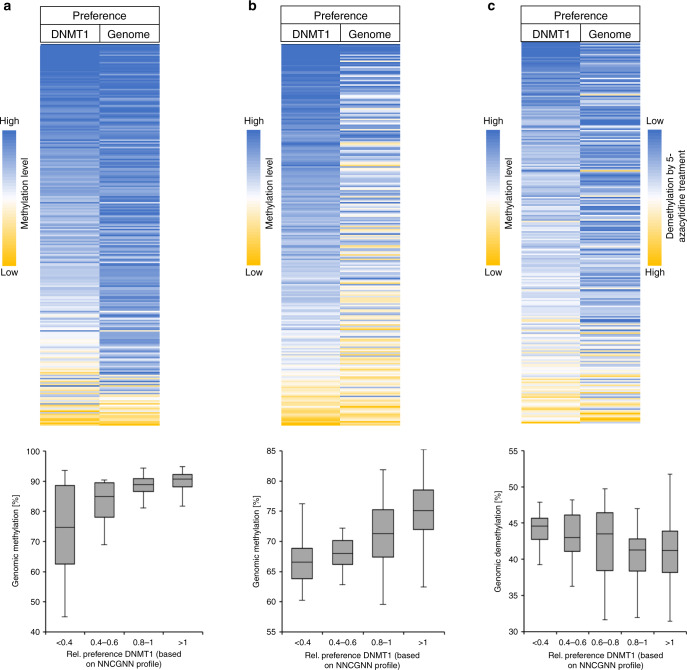
Comparison of DNMT1 flanking preferences with genomic DNA methylation. **a** Correlation of the DNMT1 flanking sequence preference (DNMT1) with genome-wide CpG methylation patterns in human cells (Genome) determined by whole genome bisulfite analysis in human ES cells^39^. The top image shows the average methylation levels of CpG sites with different NNCGNN flanks as heatmap. The *p* value of the correlation is 6.77 × 10^−13^. The lower image shows a box plot of the genomic methylation levels of CpG sites in defined ranges of DNMT1 preferences. The lines show the medians, the boxes show the 1st and 3rd quartile and the whiskers display the data maximum and minimum. **b** Correlation of the DNMT1 flanking sequence preference (DNMT1) with genome-wide CpG methylation patterns (Genome) determined by reduced representation genome bisulfite analysis in lung cancer cells^41^. The image shows the average methylation levels of CpG sites with different NNCGNN flanks as heatmaps and the corresponding box plot as described in (**a**). The *p* value of the correlation is 1.98 × 10^−10^. **c** Anticorrelation of the DNMT1 flanking sequence preference with genome-wide CpG demethylation after treatment of lung cancer cell with 5-azacytidine^41^. The image shows the DNMT1 preferences of NNCGNN flanks (DNMT1) and average levels of genome demethylation of CpG sites in NNCGNN flanks (Genome) as heatmaps and the corresponding box plot. Relative DNA demethylation is calculated as Δmethylation/initial methylation. The *p* value of the anticorrelation is 7.63 × 10^−5^. The *p* values of (anti)correlations in (**a**–**c**) were based on the Pearson correlation coefficients (*r*-factor) and the *Z*-statistics of the distribution of *r*-factors determined after randomization of one of the data sets. Source data are provided as a Source Data file.

Next we investigated if the flanking sequence preferences of DNMT1 determined in vitro influence genomic DNA methylation patterns. Charlton et al. have published a high-quality whole genome bisulfite analysis reporting the genome-wide CpG methylation in human embryonic stem (ES) cells^39^. The data provide methylation information for 20.8 million CpG sites, which we used to extract average methylation levels of CpG sites in all NNCGNN flanks. Strikingly, the genomic methylation profile determined by this approach correlated very strongly with the biochemical activity profile of DNMT1 as illustrated by the heatmaps of the methylation levels and preferences (Fig. 6a). Repetitions of the correlation analysis with randomly shuffled data revealed a *p* value of 6.8 × 10^−13^ indicating a highly significant correlation. The high congruence of both data sets is also visible when average genomic methylation levels of CpG sites are compared with preference ranges of DNMT1 (shown as box plot in Fig. 6a). This analysis revealed that average genomic methylation levels of CpG sites with flanks disfavored by DNMT1 mainly range between 62 and 88%, while average methylation levels of sites with preferred flanks mainly fluctuate between 86 and 92% clearly illustrating that the flanking sequence preferences of DNMT1 modulate genomic DNA methylation patterns.

To investigate if the dynamic changes of methylation levels in human DNA were also affected by the flanking sequence preferences, we next aimed to analyze the flanking sequence effects on DNA demethylation triggered by treatment of human cells with 5-azacytidine. This modified nucleotide is incorporated into the DNA and leads to the formation of covalent adducts with active DNMTs. Thereby, cells are depleted of DNMT1 leading to a passive loss of DNA methylation during replication^40^. We hypothesized that CpG sites disfavored by DNMT1 may lose methylation more readily under conditions of insufficient DNMT1 concentrations. We used a published reduced representation bisulfite analysis of DNA demethylation by treatment of metastatic lung cancer cells with 5-azacytidine^41^ and initially collected the average methylation levels of the untreated cells in NNCGNN flanks. As shown in Fig. 6b, a very strong correlation of genomic methylation levels with the in vitro flanking preferences of DNMT1 was observed (*p* value 1.98 × 10^−10^), similarly to what was observed in the previous analysis for ES cells. In this data set, the average methylation levels of sites with disfavored flanks mainly range from 64 to 69% while average methylation levels of sites with preferred flanks are mainly between 72 and 78%. Next, the relative level of demethylation of CpG sites by 5-azacytidine treatment was averaged for the NNCGNN flanks. This analysis showed a highly significant inverse correlation of DNA demethylation with the DNMT1 flanking sequence preferences (*p* value 7.63 × 10^−5^) (Fig. 6c) indicating that CpG sites disfavored by DNMT1 were demethylated more efficiently by 5-azacytidine treatment. Overall, these findings indicate that the flanking sequence preferences of DNMT1 shape the global DNA methylation profile and its dynamic changes in human cells. It is very striking that these effects were detectable in data sets, which aggregate the global DNA methylation profile into only 256 bins of NNCGNN sequences.

**Fig. 7:**
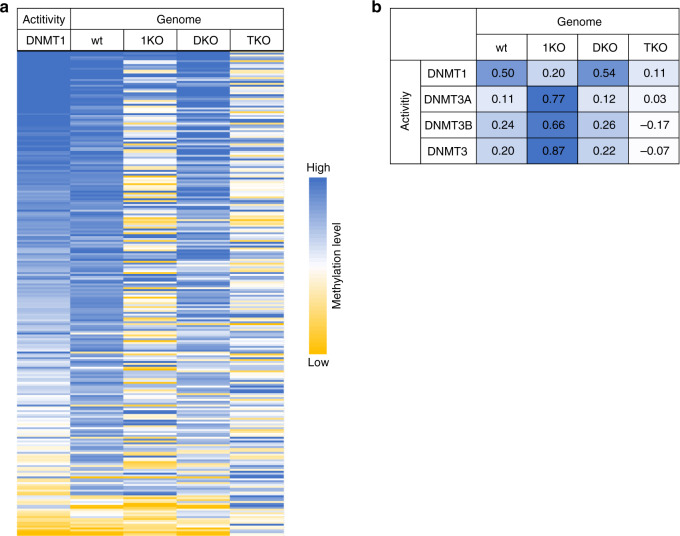
Comparison of DNMT1 flanking sequence preferences with genomic DNA methylation. **a** Correlation of the DNMT1 NNCGNN flanking sequence preferences (Activity DNMT1) with average genome-wide methylation levels of CpG sites in NNCGNN flanking contexts (Genome). Genomic methylation data were determined by whole genome bisulfite analysis in wild type mouse ES cells (wt), DNMT1 knock-out ES cells (1KO), DNMT3A/DNMT3B double knock-out ES cells (DKO) and DNMT1/DNMT3A/DNMT3B triple knock-out ES cells (TKO)^42^. The image shows the average methylation levels of individual NNCGNN sites as heatmaps. **b** Pearson correlation factors of the NNCGNN genome methylation patterns (Genome) of the different mouse ES cell lines with the flanking sequence preferences (Activity) of DNMT1, DNMT3A and DNMT3B (Fig. 3b) and an average of DNMT3A and DNMT3B (DNMT3). DNMT3A and 3B flanking preferences were taken from Gao et al.^25^.

Finally, we were aiming to confirm that the cellular methylation patterns were indeed connected to DNMT1. For this we resorted to published genome-wide DNA methylation data from wild type mouse ES cells and DNMT1 knock-out ES cells (1KO), DNMT3A/DNMT3B double knock-out ES cells (DKO) and DNMT1/DNMT3A/DNMT3B triple knock-out ES cells (TKO)^42^. The NNCGNN methylation profile analysis of these cells (Fig. 7) revealed a strong correlation of DNMT1 flanking sequence preferences with genome methylation patterns in wild type mouse ES cells (*p* = 1.9 × 10^−13^), similarly as observed in the analyses with human methylation patterns presented above. DNA methylation in DKO cells containing a double deletion of DNMT3A and DNMT3B is strongly reduced (by 78%), but the methylation pattern is correlated with the DNMT1 flanking sequence preferences even more strongly. Methylation levels in DNMT1 knock-out ES cells (1KO) are reduced to a similar degree (73%), but the residual pattern does not correlate with the DNMT1 sequence profile. Strikingly, however, the 1KO methylation profile is strongly correlated with the flanking sequence preferences of DNMT3A and DNMT3B determined in our previous work (Fig. 3b)^25^. The very low residual methylation levels in TKO cells containing a triple knock-out of DNMT1, DNMT3A and DNMT3B (reduced by 99%) did not correlate with any of the DNMT preference profiles. These findings strongly support our conclusions and document the important biological effect of flanking sequence preferences of DNMTs in the generation of biological DNA methylation patterns.

## Discussion

DNMT1-mediated maintenance DNA methylation is essential for epigenetic inheritance of DNA methylation patterns during mitotic division. Accordingly, the enzymatic activity of DNMT1 is regulated in a multifaceted fashion by many interactors and chromatin modifications^11^. Through combined kinetic, modeling and structural analyses, this work provides insights into the mechanism underlying the substrate specificity and genomic targeting of DNMT1. First, single molecule methylation profiles using a long DNA molecule with 44 hemimethylated CpG sites as substrate revealed a highly processive DNA methylation mechanism of DNMT1, and provided kinetic evidence for a conformational change of DNMT1 after binding to DNA where DNMT1 adopts a closed conformation capable of processive DNA methylation. Remarkably, we identified a strong and previously unrecognized flanking sequence preference of DNMT1. This finding motivated us to conduct a second deep enzymology experiment aiming to analyze flanking sequence preferences of DNMT1 systematically and in great detail. For this, DNA methylation of a hemimethylated CpG target site embedded into a 10 base pair random sequence context on either side was studied revealing almost 100-fold differences in methylation rates of hemimethylated CpG sites in different flanking contexts. Particularly, a disfavor for G at the −1 flank site and C at −2 was observed.

Consistently, our bioinformatic analysis of published methylome profiles determined in human ES cells and in lung cancer cells^39,41^ revealed a strong correlation between the flanking preferences of DNMT1 with modulations of methylation patterns observed in the human cells. Analyses of mouse ES cells with knock-out of different DNMTs confirmed that cellular methylation patterns in wild type cells are majorly determined by DNMT1. Moreover, we observed that the loss of DNA methylation by treatment with the demethylating agent 5-azacytidine was more pronounced at CpG sites in a flanking context disfavored by DNMT1, indicating that DNA methylation at these sites is most easily lost under conditions of insufficient amounts of DNMT1 in the cell. Overall our findings clearly indicate that the in vitro flanking sequence preferences shape the static and dynamic human methylome. Since treatment with demethylating nucleotide analogs is an established therapeutic approach^43^, the flanking sequence-dependent effects of this treatment may also be relevant in a clinical setting.

Our structural studies provide a link between the flanking sequence preferences of DNMT1 and the structural changes of the DNMT1-DNA complex at atomic level. We observed striking differences in the DNA conformation after the flipping of the target base and in the active site of DNMT1 for DNA molecules with different flanking sequences indicating flanking sequence-dependent changes in base flipping mechanisms, which is unique among the family of DNMTs. In the CCG complex, which has a very favored flanking sequence, only the target cytosine base (C7′) is flipped and only one nucleotide (C8′) shows a moderate conformational adjustment. The intrahelical positions of the nontarget strand guanine residues (G7 and G8) may be stabilized by the strong stacking of the G7–G8-A9 triade, which results from the presence of the most preferred residues at the −1 and −2 flanks. The conformational changes in the ACG complex, which has a moderately good flanking sequence, are more substantial, because the target cytosine C7′ and its Watson/Crick partner G7 are both flipped and the thymine at the −1 site in the nontarget strand (T8) shows a conformational adjustment. However, base flipping events are limited to the C7′–G7 base pair. The largest conformational changes in the DNA were observed in the original GCG complex^17^, which has an unfavorable flanking sequence. In this complex, the C7′ is flipped, its partner G7 forms a noncanonical base pair with the guanine at the −1 site in the template strand (G8′) and C8 is flipped as well. The formation of this strong G7–G8′ contact could explain the disfavor of G at the −1 site. Hence, conformational changes encompass all four nucleotides of two base pairs, the target cytosine base pair and the −1 flank base pair. The strong disfavor for a cytosine at the −2 flank site may be explained by the stabilization of the G7–G8′ base pair through stacking interactions if a guanine is present at the −2 site in the nontarget strand. This effect may also explain why the flanking sequence preferences for the −1 site were not observed in a previous study, which only varied this position^34^. Hence, the varied stability of canonical and noncanonical base pairs and strength of stacking interactions dictates the structural rearrangements of the DNA that accompany target base flipping. These effects are dependent on the flanking sequence on the minus side of the CpG site. In particular, our data link more pronounced conformational changes of the DNA to disfavored substrates. In a linear diffusion model, the methylation efficiency of a CpG site after binding of DNMT1 depends on the ratio of the rate constant for productive complex formation and the rate constant for departure of DNMT1 from the site to continue linear diffusion on the DNA. A demand for massive conformational changes may slow down the rate of an active complex formation leading to lower methylation rates at unfavorable flanks. The alternative base flipping mechanisms of DNMT1 discovered here add a new example to the large family of DNA-interacting proteins involving base flipping^44^ and may provide clues for other systems. For example, it has previously been observed that prokaryotic cytosine-C5 methyltransferases like M.HhaI (GCGC)^45^ and M.HaeIII (GGCC)^46^ follow different base flipping and rearrangement mechanisms. Our data highlight the general role of the DNA sequences surrounding the target cytosine of these enzymes on the base flipping mechanism, which can be different target sequences as in M.HhaI and M.HaeIII or different flanking sequences surrounding CpG target sites as in DNMT1.

Moreover, our study reveals a conformational transition of the catalytic helix during DNMT1-mediated DNA methylation. In the complexes with good and very good substrates (ACG and CCG), the catalytic helix predominantly adopts a kinked conformation, which has been observed in the DNA-free conformation of DNMT1^36^ and has been associated with increased activity^37^. In contrast, the catalytic helix mainly adopts a straight conformation in the complex with an unfavorable substrate (GCG)^17^. However, detailed analysis of the electron density map indicated that each of the complexes also contains a minor population of kinked/straight helix, indicating conformational dynamics of the catalytic helix during the reaction^37^. The equilibrium of these two alternative conformations appears to be influenced by the DNA conformation of the target strand (e.g. variation in the minor groove width) in particular in the segment downstream of the target C7′ at the flanking sites +1 and +2. Therefore, the flanking sequence-dependent changes in the base flipping mechanism are accompanied by conformational changes of the enzyme, which can directly influence catalysis. Interestingly, the correlation of conformational changes of the protein with flanking sequence preferences has an identical trend as observed for the DNA conformational changes; small conformational changes correlate with higher activity, presumably because active complex formation can occur faster. Our structural data suggest that the minus side region of the flanking sequence mainly determines the conformational changes of the DNA while the plus-side region influences the conformational changes of the DNMT1 protein via the MGW.

The evolutionary impact of DNA methylation has been recognized over 30 years ago with the discovery of CpG islands^47^. Later, coevolution of DNMTs with DNA repair enzymes like MBD4, Thymine DNA glycosylase and AlkB has been observed^48,49^ and phylogenetic studies revealed dynamic evolution of the DNA methylation systems with losses and gains of DNMTs and evolutionary divergence of mammalian and plant DNMTs^49–55^. Our finding that global DNA methylation patterns reflect flanking sequence preferences of DNMT1 raises the intriguing possibility that flanking sequence preferences of key methyltransferases in different species may influence their respective genomic methylation patterns. Mechanistically, the specificity of DNMT1 for methylation of hemimethylated CpG sites is increased by the binding of unmethylated CpG sites to the CXXC domains which prevents access of these substrates to the catalytic center^36^. Future studies need to investigate the flanking sequence effects on the DNMT1’s specificity to find out how preferences of the active site and putative binding preferences of the CXXC domain may act together. In a disease perspective, our data suggest that DNA demethylation by azacytidine is modulated by DNMT1 flanking sequence preferences and our previous work provided evidence that DNA methylation changes in AML patients are related to changes in the flanking sequence preference of the DNMT3A R882H mutant^24^. If and how the combined flanking sequence preferences of DNMTs and other enzymes including TET enzymes and DNA repair enzymes contribute to dynamic changes of DNA methylation in the context of cancer^56,57^, during aging^58^ or in late-replicating genomic domains^59^ remains to be elucidated.

## Materials and methods

### Expression and purification of DNMT1 for biochemical work

Full-length murine DNMT1 (UniProtKB P13864) was overexpressed and purified as described^15,18,27^ using the Bac-to-Bac baculovirus expression system (Invitrogen). In short, N-terminal His_6_- and YFP-tags were fused to the wild-type protein which was then cloned into a pFastBacHT vector. The vector was transformed into *Escherichia coli* DH10Bac cells for generation of a recombinant Bacmid followed by baculovirus amplification and DNMT1 expression in Sf21 cells. Harvested cells were lysed by sonication and the lysate was centrifuged for 1 h at 47,400*g* and 4 °C. Protein purification was performed using a column with Ni-NTA beads, including two washing steps after loading of the clear supernatant. Dialysis of the eluted DNMT1 was performed for 2 h, aliquots of the protein were frozen in liquid nitrogen and stored at −80 °C. The concentration of DNMT1 was determined using Nano-Drop (Thermo Scientific) and SDS-PAGE was used to verify the quality of the protein preparation.

### Synthesis of a 349 bp long hemimethylated substrate

The sequence of the 349 bp substrate with 44 CpG sites was taken from the CpG island upstream of the human ZNF280B (Supplementary Fig. 1a) and amplified by PCR and cloned into a plasmid (Supplementary Fig. 2). The unmethylated substrate was generated by PCR from this plasmid using a 5′-phosphorylated forward primer and a reverse primer containing 5′-phosphorothioate linkages (Primer 1 and 2 in Supplementary Table 3). The unmethylated substrate was purified using NucleoSpin® Gel and PCR Clean-up kit (Macherey-Nagel), DNA concentration was measured with Nano-Drop and agarose gel electrophoresis was used to verify the quality of the preparation. Then, the purified unmethylated substrate was methylated at 37 °C overnight with the CpG methyltransferase M.SssI in 1X NEBuffer 2.1 with 1X BSA (NEB) using 1 mM AdoMet (Sigma), 0.7 µM M.SssI and 75 ng µL^−1^ unmethylated substrate. M.SssI was expressed and purified as described^60^. The DNA methylation reaction was stopped by addition of Proteinase K (NEB) followed by incubation at 50 °C for 1 h and inactivation at 80 °C for 20 min. The substrate was purified using NucleoSpin® Gel and PCR Clean-up kit and DNA concentration was determined with Nano-Drop. Afterwards, a second methylation step was performed for 2 h using the same reaction mixture, Proteinase K treatment and purification steps. Successful methylation was verified by digestion with the CpG methylation-sensitive restriction enzyme HpaII, which only cleaves its CCGG recognition site in an unmethylated state. For the synthesis of the hemimethylated long substrate, the upper strand of the methylated substrate was digested with lambda 5′→3′ exonuclease, which preferentially cleaves DNA strands phosphorylated at the 5′-end, whereas cleavage is inhibited by phosphorothioate linkages. Therefore, the methylated substrate (final concentration of 75 ng µL^−1^) was incubated in 1× lambda exonuclease reaction buffer in the presence of 0.075 U µL^−1^ lambda exonuclease (NEB) for 2 h at 37 °C, followed by inactivation at 75 °C for 10 min. The ssDNA was then purified with NucleoSpin® Gel and PCR Clean-up kit, using NTC buffer as a special single-stranded DNA binding buffer. Nano-Drop was used to measure DNA concentration and the substrate was resolved on a 1% agarose gel to verify the digestion process. Afterwards, the hemimethylated substrate was synthesized by primer extension with the following conditions: 3 min at 95 °C, 1 min at 65 °C, 10 min at 72 °C; using a reaction mixture containing 1× Phusion® HF Buffer, 0.2 mM dNTPs, 0.08 U µL^−1^ Phusion® HF DNA Polymerase (Thermo), 0.4 µM extension primer (Primer 3 in Supplementary Table 3) and 10 ng µL^−1^ ssDNA.

### Methylation of the long hemimethylated DNA substrate

Methylation of the 349 bp long hemimethylated substrate with DNMT1 was carried out in 1X methylation buffer (100 mM HEPES, 1 mM EDTA, 0.5 mM DTT, 0.1 mg mL^−1^ BSA, pH 7.2 with KOH) in the presence of 1 mM AdoMet. For the methylation reactions, mixtures containing different DNMT1 concentrations were prepared and 18 µL aliquots were preincubated for 1 min at 37 °C. DNA methylation was started by addition of 2 µL of the hemimethylated substrate (final concentration 10 ng µL^−1^) followed by vortexing and incubation of the samples at 37 °C for different time intervals. Then, aliquots were removed and the methylation reaction stopped by freezing the mixtures in liquid nitrogen. After thawing, DNMT1 was heat inactivated for 10 min at 80 °C and the samples were bisulfite converted using the standard protocol of EZ DNA Methylation-Lightning™ Kit (ZYMO RESEARCH). Elution of the bisulfite converted DNA was performed with 10 µL of RNase free water. Bisulfite conversion levels were determined by no-enzyme controls. Cumulative methylation levels of individual substrate molecules were corrected for incomplete conversion.

### Flanking sequence preference analysis with a randomized substrate

Methylation reactions of the randomized substrate with DNMT1 were performed similarly as described for DNMT3A and DNMT3B^25^. Briefly, single-stranded oligonucleotides containing a methylated or unmethylated CpG site embedded into a 10 nucleotide random context were obtained from IDT and used for generation of 67 bps long double-stranded DNA substrates by primer extension. This pool of randomized substrates was then methylated for varying time intervals by DNMT1 using reaction mixtures with 1× methylation buffer, 1 mM AdoMet, variable amount of DNMT1 dialysis buffer to keep a fixed salt and glycerol concentration in all reactions and different DNMT1 concentrations. Reactions were stopped by freezing in liquid nitrogen. After thawing, the enzyme was inactivated for 20 min at 65 °C, hairpin ligation was conducted and bisulfite conversion was performed using EZ DNA Methylation-Lightning kit. Bisulfite conversion levels were determined by no-enzyme controls.

### Library preparation for the deep enzymology experiments

DNA libraries for Illumina NGS were prepared with a two-step PCR approach. For the long hemimethylated substrate, 1 µL of bisulfite-converted DNA was amplified in a first PCR step (PCR1) using primers containing internal barcodes (Exemplary primers 4 and 5 in Supplementary Table 3) and HotStartTaq DNA Polymerase (QIAGEN). Afterwards, the amplification efficiency was investigated by agarose gel electrophoresis. For the second amplification step (PCR2) with primers introducing adapters and indices needed for NGS (Exemplary primers 6 and 7 in Supplementary Table 3), Phusion Polymerase (Thermo) was used together with 1 µL of the sixfold diluted PCR1 products as a template. Successful amplification was verified by agarose gel electrophoresis. A DNA library of all samples pooled in equimolar amounts was purified with NucleoSpin® Gel and PCR Clean-up kit and used for Illumina sequencing.

For the randomized substrate, libraries were generated using a two-step PCR approach. Briefly, in a first PCR, 2 µL of bisulfite-converted DNA were amplified with the HotStartTaq DNA Polymerase and primers containing internal barcodes. In a second PCR, 1 µL of obtained products were amplified by Phusion Polymerase with another set of primers to introduce adapters and indices needed for NGS. The obtained libraries containing the 122 bps insert were pooled in equimolar amounts and purified using NucleoSpin® Gel and PCR Clean-up kit, followed by a second purification step of gel extraction and size exclusion with AMPure XP magnetic beads (Beckman Coulter) and used for Illumina sequencing.

### Bioinformatics analysis

NGS data sets were bioinformatically analyzed using a local instance of the Galaxy server^61^. For the long substrate, forward and reverse reads obtained from the sequencing facility as fastq files were trimmed with the software Trim Galore!, discarding sequences with a quality score below 20 (the tool was developed by Felix Krueger at the Babraham Institute). Afterwards, reads were paired using Pear^62^, filtered using Filter FASTQ tool based on the expected DNA size^63^ and mapped against a reference sequence with the bwameth tool^64^. The correctly mapped reads were demultiplexed, home written software was used to further analyze the data and final statistics were conducted with Microsoft Excel. For the randomized substrate, reads were directly trimmed and filtered according to the expected DNA size. The original DNA sequence was then reconstituted based on the bisulfite converted upper and lower strands to investigate the methylation state of the CpG site and the NNCGNN flanks. Pearson correlation factors were calculated with Excel using the correl function. *p* values were determined using the distribution of *r* values from >200 correlation analyses with one data set shuffled.

### Radioactive DNA methylation kinetics

Experimental validation of the determined flanking sequence preferences of DNMT1 was carried out using an avidin-biotin methylation plate assay^18^. For this, biotinylated double-stranded 30-mer oligonucleotides with a single hemimethylated CpG site were used. The preferred or disfavored flanking sequences were inserted into a reference substrate that was used in previous work^15,18,34^ as described in Supplementary Table 3 (Oligonucleotides 8–17) with the CpG methylation in the upper strand and biotinylation in the lower strand. Methylation with full-length mDNMT1 was conducted at 37 °C with 0.186 µM enzyme in 1× methylation buffer (100 mM HEPES, 1 mM EDTA, 0.5 mM DTT, 0.1 mg mL^−1^ BSA, pH 7.2 with KOH) in the presence of 1 µM radioactively labeled AdoMet (Perkin Elmer) and reactions were started by adding 2 µM biotinylated substrate. Reactions with mDNMT1_731–1602_ were conducted using 0.2 µM enzyme.

### Analysis of genomic DNA methylation patterns

DNA methylation in human ES cells was investigated using whole genome bisulfite data published by Charlton et al.^39^ (data set: GSM2182522_bulk_genome). Methylation levels of DNA in A549 intermediate metastatic lung cancer cell line as well as DNA demethylation by treatment with 5-azacytidine in these cells (1 mM for 6 days) were analyzed using reduced representation bisulfite sequencing data published by Hascher et al.^41^ (data sets: GSM1251240_A1R_d0 and GSM1084241_A3R_d6_1000). CpG sites were filtered for coverage ≥ 5 in both data sets in Galaxy^61^. Genome demethylation (Δ*X*) was defined by Δ*X* = (*X*_i_ – *X*_A_)/*X*_i_, with *X*_A_, genome methylation after azacytidine treatment, *X*_i_, initial genome methylation level. DNA methylation in wild type murine ES cells, as well as murine DNMT1 knock-out ES cells (1KO), DNMT3A/DNMT3B DKO and DNMT1/DNMT3A/DNMT3B triple knock-out ES cells (TKO) was investigated using whole genome bisulfite data published by Li et al. (data sets: GSM1505240-43)^42^. CpG sites were filtered for coverage > 6, only methylation data of the upper DNA strand were used. In all analyses, DNA sequences surrounding the CpG sites were retrieved using BEDTools GetFastaBed^65^. Average methylation levels in all NNCGNN flanks were determined with a home written program.

### Protein expression and purification for structural work

Expression and purification of residues 731–1602 of mouse DNMT1 (mDNMT1_731-1602_) (UniProtKB P13864) followed an established protocol^17^. In essence, the cDNA encoding mDNMT1_731–1602_ was cloned into pRSFDuet-1 vector (Novagen) containing an N-terminal His_6_-SUMO tag. The plasmid was then transformed into *E. coli* BL21 DE3 (RIL) cells. The bacterial cells were initially grown in LB medium at 37 °C. After cells density reached an *A*_600 nm_ of 0.8, protein expression was induced by addition of 0.4 mM Isopropyl β-D-1-thiogalactopyranoside (IPTG), and the cells continued to grow overnight at 15 °C. The His_6_-SUMO-mDNMT1_731-1602_ fusion protein was first purified on a Ni^2+^-NTA affinity column, followed by removal of the His_6_-SUMO tag via ULP1-mediated cleavage. Subsequently, mDNMT1_731–1602_ was sequentially purified by ion-exchange chromatography (pH 7.5) on a Heparin column (GE Healthcare) and size-exclusion chromatography (pH 7.5) on a 16/600 Superdex 200 pg column (GE Healthcare).

To generate the mDNMT1_731–1602_-DNA covalent complex, the purified mDNMT1_731-1602_ protein sample was mixed with double-stranded, hemimethylated 12-mer DNA containing a single 5fC site (for CCG DNA, upper strand: 5′-ACTTA(mC)GGAAGG-3′, lower strand: 5′-CCTTC(fC)GTAAGT-3′-; for ACG DNA, upper strand: 5′-CCTTCmCGTAAGT-3′, lower strand: 5′-ACTTA(fC)GGAAGG-3′) at a 1:2 DNMT1:DNA ratio in buffer containing 50 mM Tris-HCl (pH 7.5), 20% glycerol, 10 mM DTT and 100 µM AdoMet. The DNMT1-DNA complex was then purified through ion-exchange chromatography on a HiTrap Q XL column (GE Healthcare) and size-exclusion chromatography on a 16/600 Superdex 200 pg column (GE Healthcare). Finally, mDNM1-DNA complexes were concentrated to 0.2 mM in 20 mM Tris-HCl (pH 7.5), 250 mM NaCl, 5 mM DTT and 5% glycerol for crystallization.

### Crystallization conditions and structure determination

Crystals for mDNMT1_731–1602_-DNA complexes were generated using the hanging-drop vapor-diffusion method at 4 °C, from drops containing 0.5 µL mDNMT1_731–1602_-DNA samples and 0.5 µL precipitant solution (0.1 M Sodium Citrate pH 4.8, 10 mM ZnCl_2_). Initially, this condition yielded clusters of needle shaped crystals, which were subsequently optimized to larger plate shaped crystals by the seeding method. Crystals were soaked in cryoprotectant made of mother liquor and 25% glycerol before harvesting. The X-ray diffraction data set for the mDNMT1_731–1602_-CCG DNA complex was collected on the BL 5.0.2 beamline at the Advanced Light Source, Lawrence Berkeley National Laboratory and the diffraction data set for the mDNMT1_731–1602_-ACG DNA complex was collected on the 24-ID-C beamline at the Advanced Photon Source, Argonne National Laboratory. The diffraction data were indexed, integrated, and scaled using the HKL 3000 program^66^. The structures of the complexes were solved by molecular replacement with the PHASER^67^ program, using the structure of mDNMT1_731–1602_-ACG DNA complex (PDB 4DA4) as search model. The structural models of the mDNMT1_731–1602_-DNA complexes were then subjected to iterative modification using COOT^68^ and refinement using the PHENIX software package^69^. The same R-free test set was used throughout the refinement. The statistics for data collection and structural refinement of the mDNMT1_731-1602_-DNA complexes are summarized in Supplementary Table 4.

### Modeling of DNMT1 methylation kinetics

Two models were used to describe the kinetics of DNA methylation by DNMT1 (Fig. 2a, b). In a complex model (Model 2), DNMT1 binds to DNA (*k*_1_ and *k*_−1_) and it can undergo a conformational change into a closed conformation on the DNA (*k*_2_ and *k*_−2_). In the open state, it can methylate the DNA in a distributive reaction (${k}_{{\mathrm{met}}}^{\mathrm{d}}$), in the closed state, processive methylation (${k}_{{\mathrm{met}}}^{\mathrm{p}}$) can occur. The simplified Model 1 is a submodel of Model 2, which is obtained by setting $k_2,\,k_{ - 2}$ and $k_{{\mathrm{met}}}^{\mathrm{p}}$ to zero and considers only distributive methylation. Details of the modeling are described in Supplementary Note 1. In brief, due to the large state space, Gillespie’s algorithm^30^ was used to simulate sample paths rather than solving the Chemical Master Equation completely. Sparse grids^70^ were used to perform a first step global search for parameter estimation, from which sets of good and diverse parameters were selected as starting points for multi-start local optimization. AIC^71^ was used for model comparison.

### Reporting summary

Further information on research design is available in the Nature Research Reporting Summary linked to this article.

## Supplementary information

Supplementary Information

Peer Review

Reporting Summary

## Data Availability

The data that support this work are available from the corresponding authors upon reasonable request. Structural data have been deposited with the PDB with accession codes 6W8W [10.2210/pdb6W8W/pdb] and 6W8V [10.2210/pdb6W8V/pdb]. DNMT1 NGS kinetic raw data have been uploaded at DaRUS, the Data Repository of the University of Stuttgart [10.18419/darus-628] and [10.18419/darus-629]. Source data are provided with this paper.

## Source data

Source Data

## Code availability

The code for kinetic modeling is accessible at https://github.com/TheVincentWagner/SimModelDNAMethylation.git and directly executable with a python 3 distribution. All additional scripts used in this study for data analysis of the NGS kinetics are available upon request. Source data are provided with this paper.
