# Supplementary material for: DNA sequence-dependent activity and base flipping mechanisms of DNMT1 regulate genome-wide DNA methylation
Source: Nat Commun. 2020 Jul 24;11:3723. doi: 10.1038/s41467-020-17531-8 (PMC7381644; doi:10.1038/s41467-020-17531-8)
Supplement: Supplementary file 1 — Supplementary Information [file 41467_2020_17531_MOESM1_ESM.pdf]

DNA sequence-dependent activity and base flipping mechanisms of DNMT1 regulate genome-wide DNA methylation

Adam et al.

Supplementary information

Supplementary Figures

Supplementary Figure 1: Workflow for the deep enzymology experiments conducted in this study.

Supplementary Figure 2: Preparation of the hemimethylated 349 bp substrate with 44 CpG sites.

Supplementary Figure 3: Technical controls and quality information regarding the random flank library substrate methylation experiments.

Supplementary Figure 4: Additional controls regarding the random flank library substrate methylation experiments.

Supplementary Figure 5: Crystal structures of the CCG and ACG complexes of mDNMT1.

Supplementary Figure 6: Molecular details of the mDNMT1$_{731-1602}$-DNA interactions.

Supplementary Figure 7: Alternative conformation of the catalytic helix of mDNMT1731-1602 in the ACG and CCG complexes.

Supplementary Figure 8: Correlation of minor groove width with DNMT1 methylation activities.

Supplementary Tables

Supplementary Table 1: Summary of the NGS data obtained with the 349 bp substrate.

Supplementary Table 2: Summary of the NGS data obtained with the hemimethylated substrate with randomized flanks.

Supplementary Table 3: Summary of the primers and oligonucleotides used during this work.

Supplementary Table 4: X-ray data collection and refinement statistics.

Supplementary Note

Supplementary Note 1: Detailed method description for modeling of DNMT1 reaction kinetics.

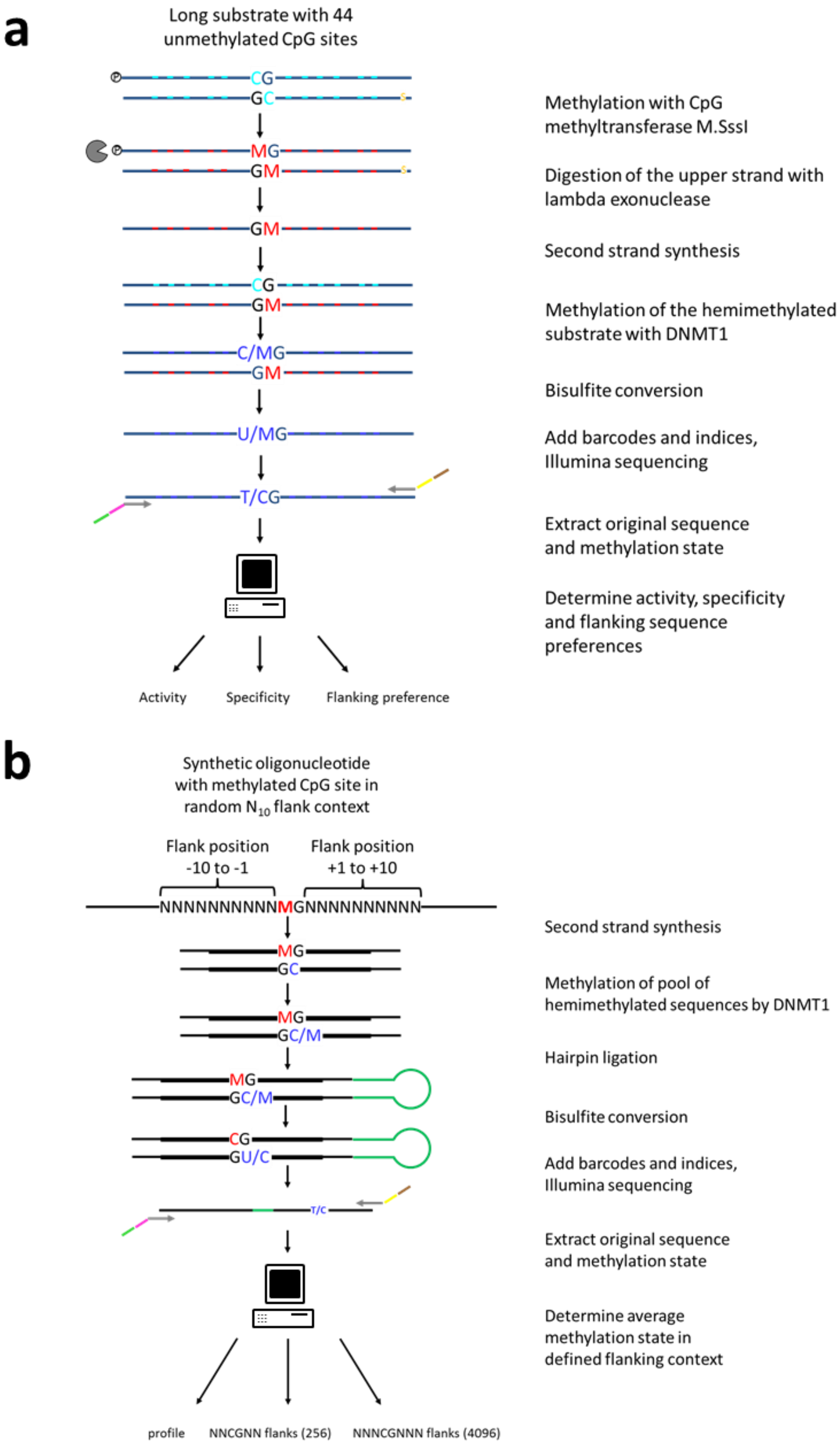

Supplementary Figure 1: Workflow for the deep enzymology experiments conducted in this study. a) Workflow for the generation and methylation analysis of the hemimethylated 349 bp substrate. **b)** Workflow for the generation and methylation analysis of the library of substrates with a hemimethylated CpG site in a context of 10 randomized base pairs on either side.

a

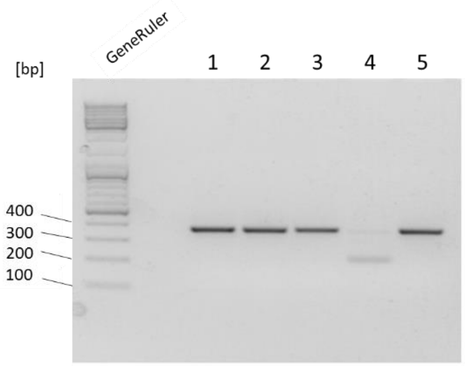

b

```
                                     1          2  3   4     5             6               7 8       9     10          11
5′-CTTGCCATGAAATCCCTCAAGTCTCCCCTCAGGGAACGCTGAAGCCGCGCCACGCCCCCGTCCTTACCAGTCCGGATCAGCTGCTGTTCGCGAGCTGCCGGCCACGCACCAGCCCCG
3′-GAACGGTACTTTAGGGAGTTCAGAGGGGAGTCCCTTGCGACTTCGGCGCGGTGCGGGGGCAGGAATGGTCAGGCCTAGTCGACGACAAGCGCTCGACGGCCGGTGCGTGGTCGGGGC

    12    13          14 15 16    17              18 19 20 21 22 23 24       25 26 27 28             29                30
GAGGCGCTCCCGGGGCACAGCCGGCGGCGACTACGCCTCCTCAGGCCCCCGGCGCCGCCGACGCGCACGCCTCCACACGCGCGCGTCCAGTGGAGACCTGCGATTGGCTGCCAGGTGCCG
CTCCGCGAGGGCCCCGTGTCGGCCGCCGCTGATGCGGAGGAGTCCGGGGGCCGCGGCGGCTGCGCGTGCGGAGGTGTGCGCGCGCAGGTCACCTCTGGACGCTAACCGACGGTCCACGGC

 31 32  33 34 35    36            37 38 39     40          41 42        43 44
GCGCGAGATCGGCGCGGCTCCGAGCTAGGAGCATGCGCGCGCTCTGACGCCCCTGGTGGCGACGGCTGGACGCGGGGTTAAATTGAGAAGGAGGAGGGCAGCAGCAATACCC-3′
CGCGCTCTAGCCGCGCCGAGGCTCGATCCTCGTACGCGCGCGAGACTGCGGGGACCACCGCTGCCGACCTGCGCCCCAATTTAACTCTTCCTCCTCCCGTCGTCGTTATGGG-5′
```

Supplementary Figure 2: Preparation of the hemimethylated 349 bp substrate with 44 CpG sites. a) Quality control steps of substrate preparation. The figure shows an exemplary agarose gel stained with GelRed showing the unmethylated PCR product (1), sample 1 after methylation with M.SssI (2), sample 2 after a second methylation reaction with M.SssI (3), sample 3 after treatment with lambda-exonuclease (4), and sample 4 after second strand synthesis (5). **b)** Sequence of the 44-sites substrate with the CpG sites highlighted. Source data are provided as a Source Data file.

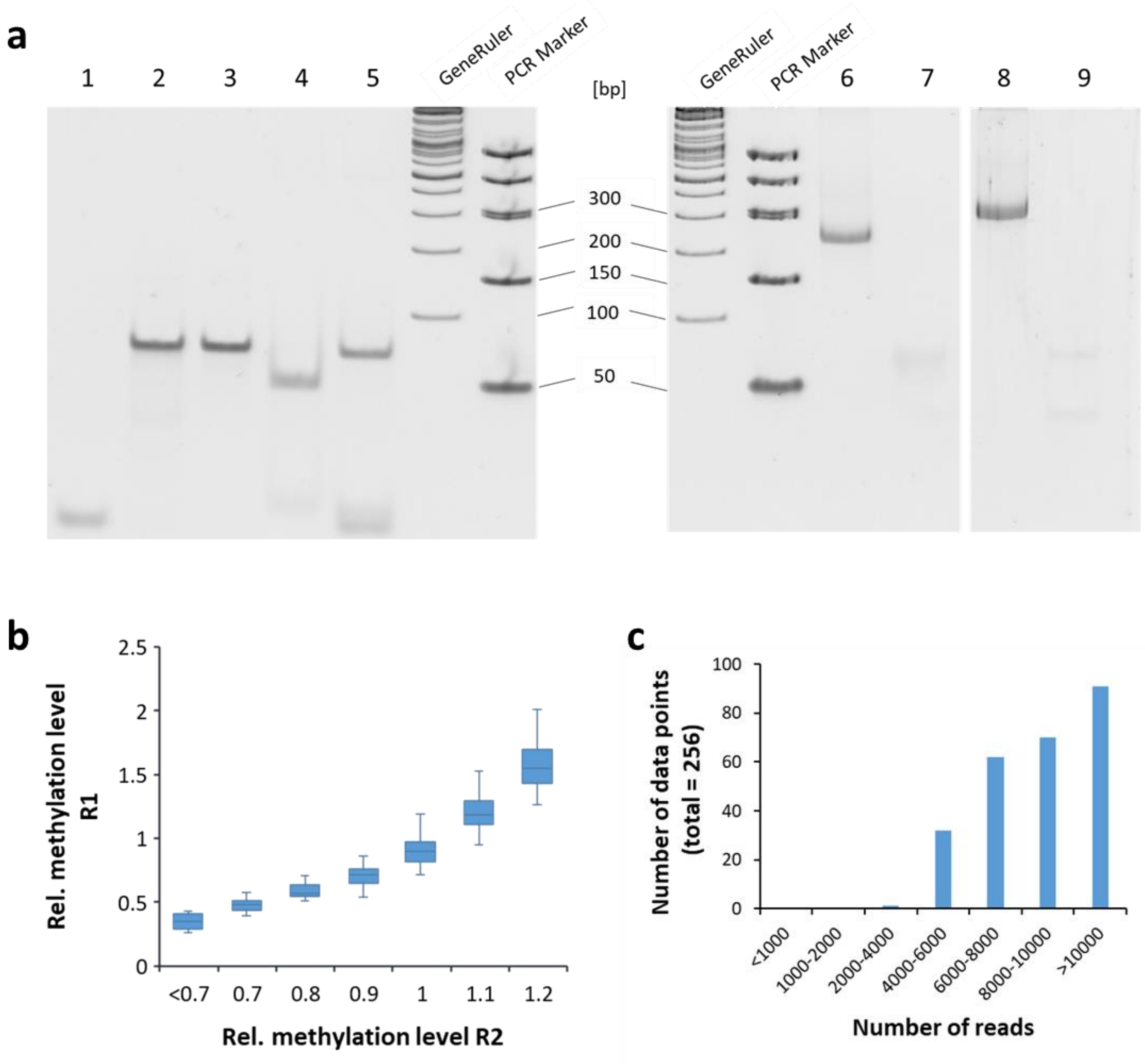

Supplementary Figure 3: Technical controls and quality information regarding the random flank library substrate methylation experiments. a) Quality control steps for the preparation of the hemimethylated substrate with one CpG site in randomized sequence context and library generation. The figure shows exemplary 10% polyacrylamide gels stained with GelRed showing the hairpin (1), double-stranded DNA after second strand synthesis using the synthetic oligonucleotide as template (2), sample 2 purified (3), sample 3 digested with BsaI-HF v2 (4), sample 4 after ligation of the hairpin (5), PCR1 product with added barcodes after bisulfite conversion of 5 (6), non-template control for PCR1 (7), PCR2 product with added indices (8), and the non-template control for PCR2 (9). Samples 6-9 were analyzed on the same gel. **b)** Box plot showing the relative methylation levels at NNCGNN sites observed in the experimental repeats 1 and 2. Data were normalized to the average methylation. Methylation levels in repeat 2 were clustered in bins and the corresponding methylation levels in repeat 1 are shown as displayed. The lines show the medians, the boxes show the 1st and 3rd quartile and the whiskers display the data maximum and minimum. **c)** Total number of NGS sequence reads in the NNCGNN bins showing an ultra-deep coverage. Source data are provided as a Source Data file.

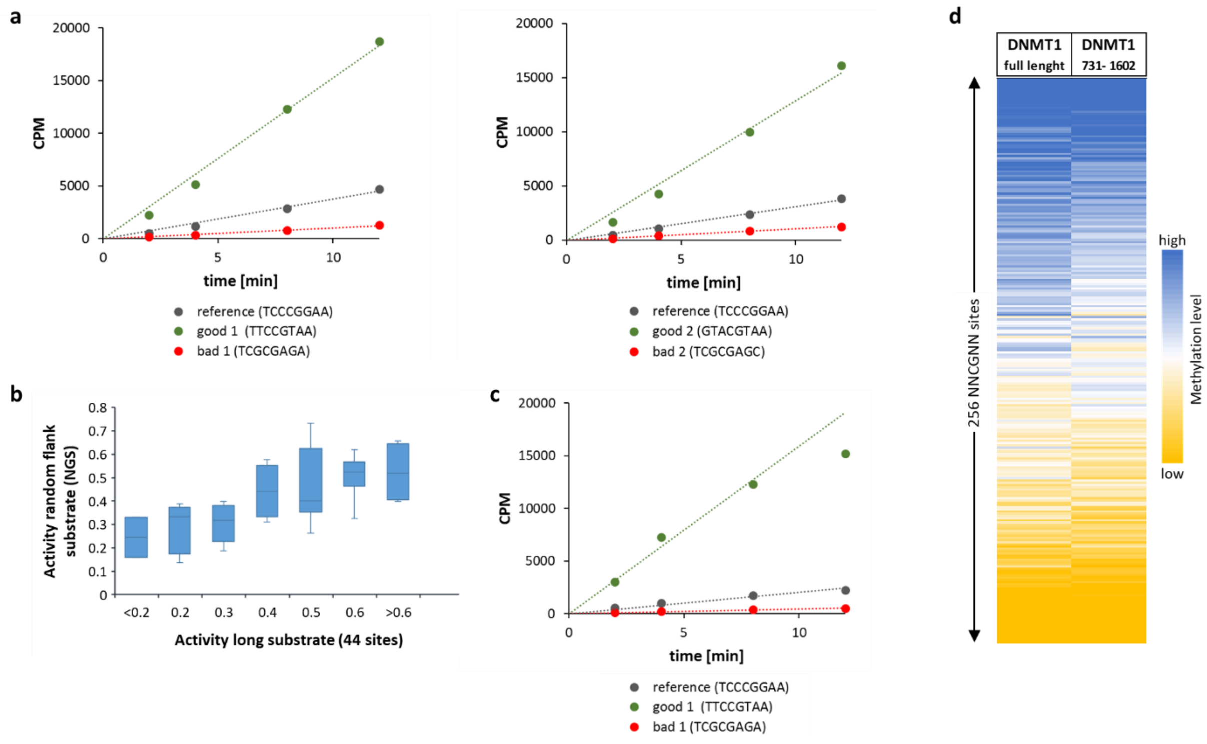

Supplementary Figure 4: Additional controls regarding the random flank library substrate methylation experiments. a) Example of kinetic data for the methylation of two pairs of substrates designed to be preferred or disfavored by DNMT1 in comparison with a reference substrate that has been used in previous studies and is mildly disfavored. These experiments were conducted with full-length mDNMT1. Average results and standard deviations of repeated methylation experiments are reported in Table 1. **b)** Box plot showing the correlation of methylation rates determined for the 44 CpG sites on the long substrate with the rates determined for the same sites in the random flank library NGS methylation experiment. The lines show the medians, the boxes show the 1st and 3rd quartile and the whiskers display the data maximum and minimum. **c)** Example of kinetic data for the methylation of the substrate pair shown in panel a (left) with the mDNMT1(731-1602) fragment used for the structural analysis. **d)** Comparison of the flanking sequence preferences of full-length mDNMT1 and mDNMT1(731-1602). The methylation levels of NNCGNN sites are displayed as heatmaps showing the strong correlation of both data sets (Pearson correlation factor r=0.89). Source data are provided as a Source Data file.

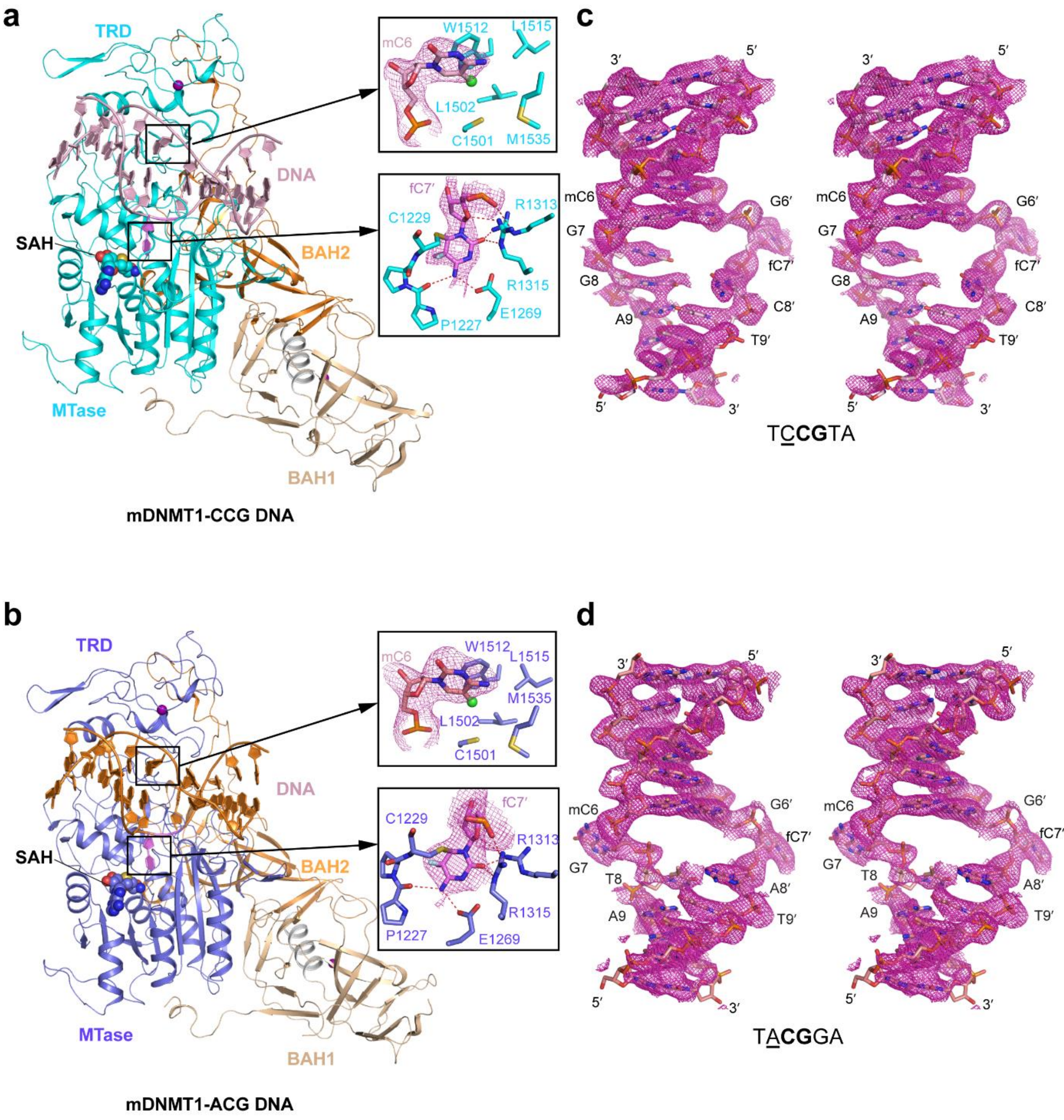

Supplementary Figure 5: Crystal structures of the CCG and ACG complexes of murine DNMT1. a, b) Ribbon representation of mDNMT1$_{731\text{-}1602}$ covalently bound to 5fC-containing CCG (a) and ACG (b) DNA. The BAH1 and BAH2 domains are colored wheat and orange, respectively. The MTase domain is colored in cyan in the CCG complex (a) and slate in the ACG complex (b). The domain linker between the BAH1 and BAH2 domains is colored grey. The DNMT1-bound DNAs are colored light pink and orange, respectively. Zinc ions and the flipped-out fC7' are purple and violet, respectively. The AdoHcy (SAH) molecule is in sphere representation. DNMT1 residues interacting with mC6 and fC7' are shown in expanded views in both (a) and (b). The Fo-Fc omit maps for mC6 and fC7' are shown as magenta mesh, contoured at 2.0 σ level, in the expanded views. The hydrogen bonds are shown as dashed lines. **c, d)** Stereo views of the Fo-Fc omit maps for the DNMT1-bound CCG (c) and ACG (d) DNA shown as magenta mesh contoured at 1.3 σ level. The -1 flanking bases on the target strand of both DNA molecules show lower electron density than the neighboring bases at elevated contour level (not shown), indicative of higher dynamic property.

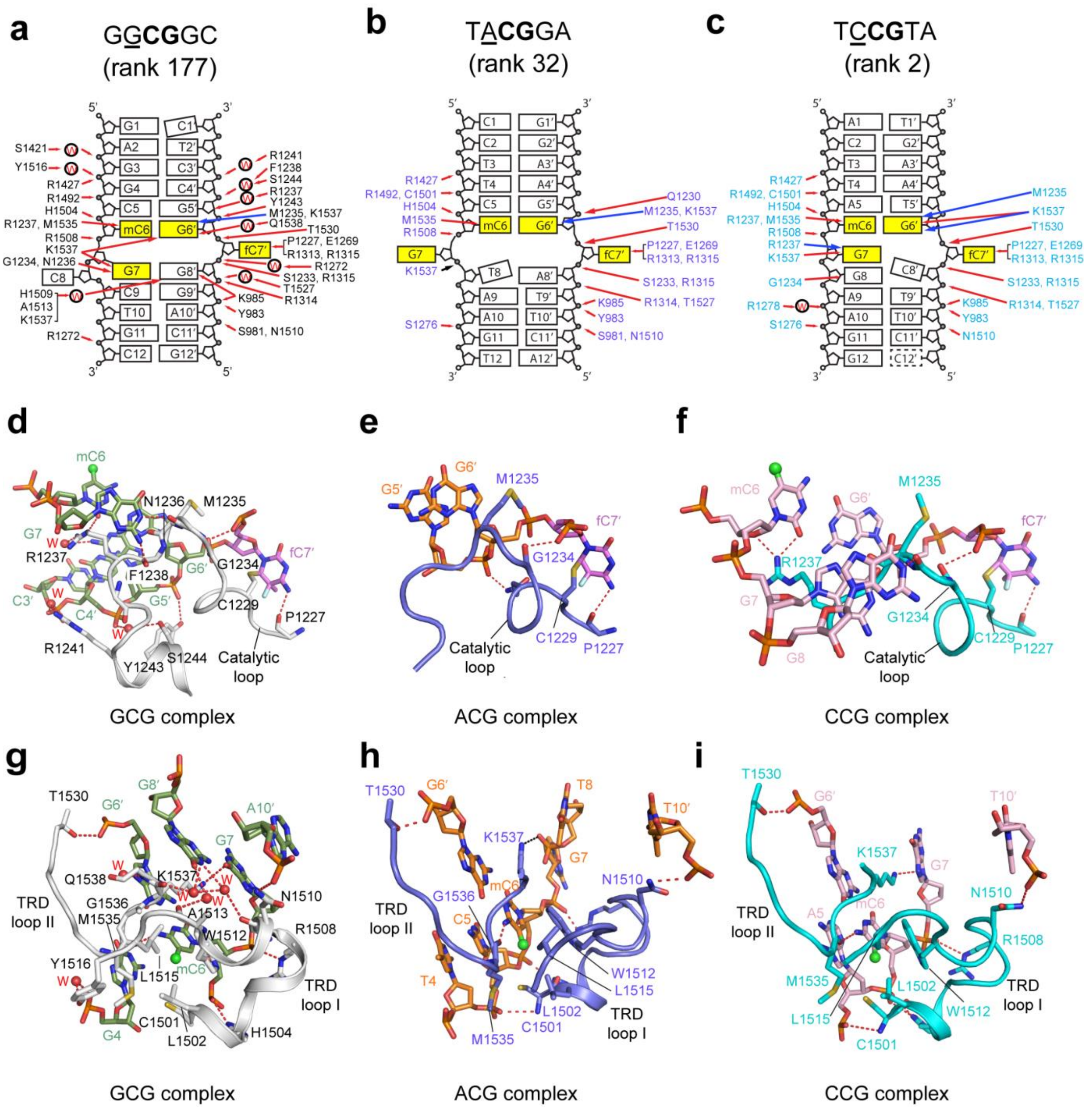

Supplementary Figure 6: Molecular details of the mDNMT1$_{731\text{-}1602}$-DNA interactions. a-c) Schematic views of the protein-DNA interactions in the GCG complex (a), adopted from a previous report (PDB 4DA4)[1], ACG (b) and CCG (c) complexes. The hydrogen bonding and base-stacking contacts are indicated by red and blue arrows, respectively. Water-mediated hydrogen bonds are labeled as letter 'W' inside a circle. In the CCG complex (c), the base of C12' is indicated by a dashed box due to missing of electron density. **d-f)** Close-up views of the catalytic loop-DNA interactions in the GCG complex (PDB 4DA4) (d), ACG complex (e) and CCG complex (f). **g-i)** Close-up views of the interactions between the TRD loops and DNA in the GCG complex (PDB 4DA4)[1] (g), ACG complex (h), and CCG complex (i). The hydrogen bonds are shown as dashed lines in red. Water molecules are shown as red spheres. The 5-methyl group in mC6 is shown as green sphere. The electrostatic interaction is shown as dashed line in black in (h).

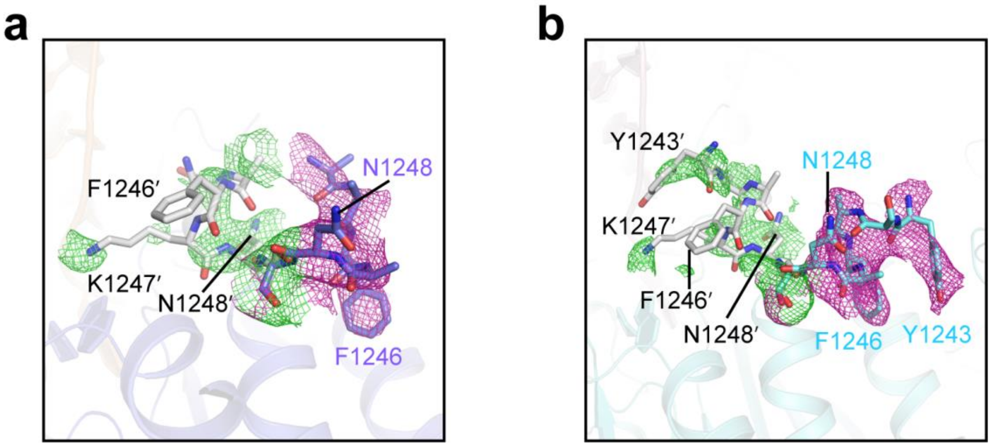

Supplementary Figure 7: Alternative conformation of the catalytic helix of mDNMT1$_{731\text{-}1602}$ in the ACG and CCG complexes. (a,b) Fo-Fc omit maps of the two alternative conformations of the mDNMT1$_{731\text{-}1602}$ in the ACG (a) and CCG (b) complexes, contoured at 2.0 σ level. The straight conformations and associated maps are colored in silver and green, respectively. The kinked conformations are colored in slate and aquamarine in the ACG and CCG complexes, respectively, and their associated maps are colored in magenta. Residues for the minor conformations are labeled with prime.

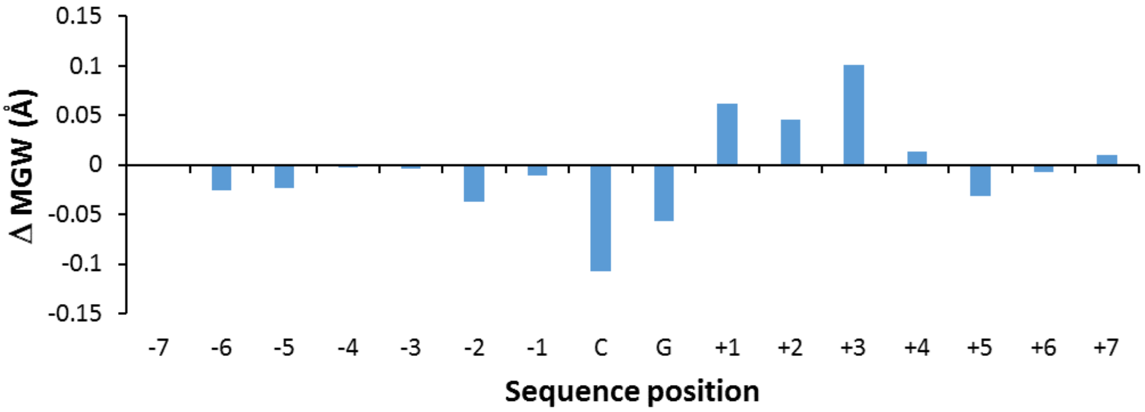

Sequence position	-7	-6	-5	-4	-3	-2	-1	C	G	+1	+2	+3	+4	+5	+6	+7
p-value	0.27	0.18	0.23	0.31	0.33	0.06	0.49	2.5E-09	2.8E-03	5.4E-06	3.3E-04	5.6E-12	0.07	0.10	0.40	0.11

Supplementary Figure 8: Correlation of minor groove width (MGW) with DNMT1 methylation activities. Minor groove width was calculated with the DNA shape prediction server for all pentanucleotides at each position of the DNA (http://rohslab.cmb.usc.edu/DNAshape/). Afterwards at each position the average MGWs was determined for the methylated molecules and for all molecules and the difference of both numbers calculated (Δ MGW). Two critical regions were detected: 1) the CpG base pairs where the MGW of methylated sequences are reduced and 2) the +1 to +3 flank, where the MGW of methylated sequence is increased. Position specific p-values were calculated using Z-statistics on the basis of the distribution of Δ MGW values at the -7 to -1 and +4 to +7 positons, where no strong effects were observed.

Supplementary Table 1: Summary of the NGS data obtained with the 349 bp substrate.

Experiment	Substrate	c(DNMT1) [µM]	Incubation time [min]	No. of reads	Methylation level
Repeat 1	hemimethylated upper strand	0.186	1	55093	0.104
	hemimethylated upper strand	0.186	3	54749	0.208
	hemimethylated upper strand	0.186	10	74123	0.508
	hemimethylated upper strand	0.186	30	69361	0.690
Repeat 2	hemimethylated upper strand	0.186	1	55530	0.109
	hemimethylated upper strand	0.186	3	53508	0.226
	hemimethylated upper strand	0.186	10	61537	0.517
	hemimethylated upper strand	0.186	30	61127	0.682
Controls	hemimethylated upper strand	-	-	12069	0.018
	hemimethylated lower strand	-	-	2974	0.984
	unmethylated upper strand	-	-	58514	0.004

As control, the methylation state of the hemimethylated and unmethylated substrates was determined.

Supplementary Table 2: Summary of the NGS data obtained with the hemimethylated substrate with randomized flanks.

Experiment	Substrate	c(DNMT1) [µM]	Incubation time [min]	No. of reads	Methylation level
Repeat 1	hemimethylated	0.021	12	2227342	0.131
Repeat 2	hemimethylated	0.062	12	134713	0.777
Controls	unmethylated with enzyme	0.062	12	170870	0.0047
	hemimethylated without enzyme	-	-	150155	0.0030
	unmethylated without enzyme	-	-	143836	0.0028

As controls, methylation of an unmethylated substrate and the methylation of the substrate without enzyme treatment was determined.

Supplementary Table 3: Summary of the primers and oligonucleotides used in this work.

No.	**Sequence**
1	pCCTGCCATGAAATCCCTCAAGTCTC
2	GGGTATTGCTGCTGCCCTC
3	CCTGCCATGAAATCCCTCAAGTCTC
4	ACACTCTTTCCCTACACGACGCTCTTCCGATCTNNNNNAGAGCTTTGTTATGAAATTTTTTAAGTTTTT TTTTAGG
5	GTGACTGGAGTTCAGACGTGTGCTCTTCCGATCTNNNNNAGCATAAATATTACTACTACCCTCCTCCT TCTCAATTTAAC
6	AATGATACGGCGACCACCGAGATCTACACATTCAGAAACACTCTTTCCCTACACGACGCTCTTCCGAT CT
7	CAAGCAGAAGACGGCATACGAGATTTCTGAATGTGACTGGAGTTCAGACGTGTGCTCTTCCGATCT
8	Biotin-TTGCACTCTCCTTCCGTAAGTCCCAGCTTC
9	GAAGCTGGGACTTA^{m}CGGAAGGAGAGTGCAA
10	Biotin-TTGCACTCTCCTCGCGAGAGTCCCAGCTTC
11	GAAGCTGGGACTCT^{m}CGCGAGGAGAGTGCAA
12	GAAGCTGGGACTTC^{m}CGGGAGGAGAGTGCAA
13	Biotin-TTGCACTCTCCTCCCGGAAGTCCCAGCTTC
14	Biotin-TTGCACTCTCCTCGCGAGCGTCCCAGCTTC
15	GAAGCTGGGACGCT^{m}CGCGAGGAGAGTGCAA
16	Biotin-TTGCACTCTCCGTACGTAAGTCCCAGCTTC
17	GAAGCTGGGACTTA^{m}CGTACGGAGAGTGCAA

Exemplary barcode and index parts are underlined. N refers to random nucleotides.

Supplementary Table 4: X-ray data collection and refinement statistics.

	**mDNMT1–CpCpG DNA (PDB: 6W8W)**	**DmNMT1–ApCpG DNA (PDB: 6W8V)**
**Data collection**		
Space group	P 1 2_1 1	P 1 2_1 1
Cell dimensions		
a, b, c (Å)	89.8, 152.6, 95.6	89.2, 152.1, 96.0
α, β, γ (°)	90, 94.7, 90	90, 94.3, 90
Resolution (Å)	45.5-3.0(3.1-3.0)[a]	47.9-3.1(3.2-3.1)[a]
R_{merge}	0.331(1.714)	0.395(2.05)
$I/\sigma(I)$	4.2(0.6)	4.1(0.6)
$CC_{1/2}$	0.974(0.302)	0.981(0.329)
Completeness (%)	97.9(93.0)	99.0(91.1)
Redundancy	6.6(5.5)	6.7(5.9)
Refinement		
No. Reflections	50,786	45,085
R_{work} / R_{free}	0.238/0.280	0.246/0.278
No. Atoms		
Protein and DNA	13,821	13,899
AdoHcy	52	52
Zn^{2+}	4	4
Water	64	
B factors ($Å^2$)		
Protein	82.9	77.9
DNA	132.0	116.8
Zn^{2+}	93.4	70.8
AdoHcy	81.8	82.4
Water	50.0	
r.m.s deviations		
Bond lengths (Å)	0.003	0.003
Bond angles (°)	0.651	0.627

[a]Values in parentheses are for highest-resolution shell. Each structure was determined using the dataset collected from a single crystal.

Supplementary Note 1: Detailed method description for modeling of DNMT1 reaction kinetics

Stochastic modeling

The Chemical Master Equation (CME) describes the dynamics of the probability distribution over all possible microstates of a chemical reaction system[2]. In our case, a microstate is specified by the methylation patterns of all DNA molecules and the binding states of the DNMT1 molecules. We encode the methylation pattern at time t with a matrix $\mathbf{M} \epsilon \{0,1\}^{44 \times n_{\mathrm{DNA}}}$, which assigns Boolean values to each of the 44 CpG sites on every of the n_{DNA} DNA molecules and indicates for each site whether it is methylated or not. A DNMT1 molecule j can be in three different states $x_{\mathrm{j}} \epsilon \{z_0, z_1^{\mathrm{i}}, z_2^{\mathrm{i}}\}$, where z_0, z_1^{i} and z_2^{i} denote unboundedness and boundedness to DNA molecule i in open and closed conformation, respectively. The dependence of the methylation reaction propensities $a(x_{\mathrm{j}}, \mathbf{M}_{\mathrm{i}})$ on the methylation pattern $\mathbf{M}_{\mathrm{i}} \epsilon \{0,1\}^{44}$ of the respective DNA molecule i is required to keep track of these patterns, resulting in a large state space. Hence, we used Gillespie's algorithm[3] to simulate sample paths rather than solving the CME completely.

Our full kinetic model (Model 2) comprises six chemical reactions. DNMT1 binds to DNA into an open state conformation (k_1 and k_{-1}) and it can undergo a conformational change into a closed conformation on the DNA (k_2 and k_{-2}). In the open state, it can methylate the DNA in a distributive reaction ($k_{\mathrm{met}}^{\mathrm{d}}$), while in the closed state processive methylation ($k_{\mathrm{met}}^{\mathrm{p}}$) can occur. The methylation rate constants were defined to combine the two elementary steps of methylation and either dissociation from the DNA ($k_{\mathrm{met}}^{\mathrm{d}}$) or movement to the next methylation site ($k_{\mathrm{met}}^{\mathrm{p}}$), because these steps could not be separated on the basis of the available experimental data. We assumed that DNMT1 molecules do not directly interact with each other, which allows to simulate each DNMT1 molecule separately one after another:

<u>Reaction 1:</u> $z_0 \overset{k_1}{\rightarrow} z_1^{\mathrm{i}}$, association of DNMT1 to DNA, which is assumed to be of zero order with respect to the number of DNA.

propensity $a_1(x_{\mathrm{j}}) = \begin{cases} k_1 & if\ x_{\mathrm{j}} = z_0 \\ 0 & else \end{cases}$ (1)

<u>Reaction 2:</u> $z_1^{\mathrm{i}} \overset{k_{-1}}{\longrightarrow} z_0$, dissociation of DNMT1 from DNA.

propensity $a_{-1}(x_{\mathrm{j}}) = \begin{cases} k_{-1} & if\ x_{\mathrm{j}} = z_1^{\mathrm{i}} \\ 0 & else \end{cases}$ (2)

<u>Reaction 3:</u> $z_1^{\mathrm{i}} \overset{k_{\mathrm{met}}^{\mathrm{d}}}{\longrightarrow} z_0 + \mathrm{methylation}$, distributive methylation as an effective reaction comprising methylation and dissociation.

propensity $a_{\mathrm{d}}(x_{\mathrm{j}}, \mathbf{M}_{\mathrm{i}}) = \begin{cases} k_{\mathrm{met}}^{\mathrm{d}} * \alpha(\mathbf{M}_{\mathrm{i}}) & if\ x_{\mathrm{j}} = z_1^{\mathrm{i}} \\ 0 & else \end{cases}$ (3)

<u>Reaction 4:</u> $z_1^{\mathrm{i}} \overset{k_2}{\rightarrow} z_2^{\mathrm{i}}$, conformational change of DNMT1 into closed conformation, in which it is tightly bound to the DNA.

propensity $a_2(x_j) = \begin{cases} k_2 & if\ x_j = z_1^i \\ 0 & else \end{cases}$ (4)

Reaction 5: $z_2^i \xrightarrow{k_{-2}} z_1^i$, reverse conformational change.

propensity $a_{-2}(x_j) = \begin{cases} k_{-2} & if\ x_j = z_2^i \\ 0 & else \end{cases}$ (5)

Reaction 6: $z_2^i \xrightarrow{k_{met}^p} z_2^i + \text{methylation}$, processive methylation.

propensity $a_p(x_j, \mathbf{M}_i) = \begin{cases} k_{met}^p * \alpha(\mathbf{M}_i) & if\ x_j = z_2^i \\ 0 & else \end{cases}$ (6)

The factor $\alpha(\mathbf{M}_i) \epsilon (0,1)$ was defined in the following way: First, the methylation rates of the 44 CpG sites in Figure 1d were normalized to obtain a probability distribution:

Site	1	2	3	4	5	6	7	…	42	43	44
Probability	0.021	0.032	0.005	0.006	0.019	0.052	0.039	…	0.033	0.025	0.01

These probabilities were stored in a vector $\mathbf{A} \epsilon \mathbb{R}^{44}$ and $\alpha(\mathbf{M}_i)$ was calculated as

$$\alpha(\mathbf{M}_i) = 1 - \mathbf{A}^T \mathbf{M}_i \ (7)$$

This takes into account that a methylation is more likely if less sites are methylated.

The CpG site to be methylated was chosen according to the probabilities of the yet unmethylated sites, which were therefore rescaled to a proper probability distribution.

Pseudocode SSA Model 2

Init $t = 0$, parameters $\theta = (k_1, k_{-1}, k_2, k_{-2}, k_{met}^d, k_{met}^p)$, $\mathbf{M}(t = 0) = 0^{44 \times n_{DNA}}$,

for every DNMT1 protein $j = 1 \dots n_{DNMT1}$ do

  $x_j(t = 0) = z_0$ (DNMT1 initially unbound)

  while ($t < t_{end}$) do

  calculate propensities $a_1(x_j), a_{-1}(x_j), a_2(x_j), a_{-2}(x_j), a_d(x_j, \mathbf{M}_i)$ and $a_p(x_j, \mathbf{M}_i)$

    If $x_j(t) = z_0$

      calculate $a_{sum}(x_j) = a_1(x_j)$

      set reaction type $r = 1$, choose i uniformly distributed, $I \sim DU(1, n_{DNA})$

    Else if $x_j(t) = z_1^i$ for any DNA molecule i

      calculate $a_{sum}(x_j, \mathbf{M}_i) = a_{-1}(x_j) + a_2(x_j) + a_d(x_j, \mathbf{M}_i)$

      draw next reaction type $r \epsilon \{-1, 2, d\}$, $R \sim \frac{a_r(x_j, \mathbf{M}_i)}{a_{sum}(x_j, \mathbf{M}_i)}$

Else if $x_{\mathrm{j}}(t) = z_2^{\mathrm{i}}$ for any DNA molecule i

calculate $a_{\mathrm{sum}}(x_{\mathrm{j}}) = a_{-2}(x_{\mathrm{j}}) + a_{\mathrm{p}}(x_{\mathrm{j}}, \mathbf{M}_{\mathrm{i}})$

draw next reaction type r ϵ $\{-2, \mathrm{p}\}$, R $\sim \frac{a_{\mathrm{r}}(x_{\mathrm{j}}, \mathbf{M}_{\mathrm{i}})}{a_{\mathrm{sum}}(x_{\mathrm{j}}, \mathbf{M}_{\mathrm{i}})}$

Draw waiting time $\tilde{t}$ to next reaction, $\tilde{T} \sim \mathrm{Exp}\left(a_{\mathrm{sum}}(x_{\mathrm{j}})\right)$

Update state $x_{\mathrm{j}}(t)$ and $\mathbf{M}$ by performing reaction r and set $t = t + \tilde{t}$

Model 1 is a submodel of Model 2, which is obtained by setting k_2, k_{-2} and $k_{\mathrm{met}}^{\mathrm{p}}$ to zero.

Parameter Estimation

The parameters of our model are $\theta_1 = (k_1, k_{-1}, k_{\mathrm{met}}^{\mathrm{d}})$ for Model 1 and $\theta_2 = (k_1, k_{-1}, k_2, k_{-2}, k_{\mathrm{met}}^{\mathrm{d}}, k_{\mathrm{met}}^{\mathrm{p}})$ for Model 2. We used sparse grids[4] in a first step to perform a global search, from which we selected sets of good and diverse parameters as starting points for multi-start local optimization. We used scipy.optimize.fmin_cobyla with the constraint $k_{\mathrm{met}}^{\mathrm{d}} < k_{\mathrm{met}}^{\mathrm{p}}$ for Model 2. This constraint takes into account that the distributive methylation reaction actually combines methylation and physical dissociation from the DNA, resulting in the expectation of a smaller effective rate. The number of DNA molecules was $n_{\mathrm{DNA}} = 10^3$ during the optimization, but was increased to 2.5×10^4 for model comparison and to generate results presented in Figure 2. The number of DNMT1 proteins n_{DNMT1} was always chosen to be four times larger than n_{DNA}. The code is accessible at https://github.com/TheVincentWagner/SimModelDNAMethylation.git and directly executable with a python 3 distribution.

Model comparison

Akaike's Information Criterion (AIC)[5] was used for model comparison. A lower AIC value indicates a better model, and

$$\exp\left(\frac{\mathrm{AIC(Model\ 2)} - \mathrm{AIC(Model\ 1)}}{2}\right) = \exp\left(\frac{-738.38 + 437.63}{2}\right) = \exp(-150.375) \quad (8)$$

indicates that Model 2 is by a factor $\exp(150)$ better suited to explain the experimental data, which confirms a highly significant preference for Model 2.

Supplementary References

1 Song, J., Teplova, M., Ishibe-Murakami, S. & Patel, D. J. Structure-based mechanistic insights into DNMT1-mediated maintenance DNA methylation. *Science* **335**, 709-712 (2012).

2 Higham, D. J. Modeling and simulating chemical reactions. *Siam Rev* **50**, 347-368 (2008).

3 Gillespie, D. T. General Method for Numerically Simulating Stochastic Time Evolution of Coupled Chemical-Reactions. *J Comput Phys* **22**, 403-434 (1976).

4 Garcke, J. & Griebel, M. in *Lecture Notes in Computational Science and Engineering* Vol. 88 x, 276 pages (Springer Science & Business Media, 2012).

5 Burnham, K. P., Anderson, D. R. & Huyvaert, K. P. AIC model selection and multimodel inference in behavioral ecology: some background, observations, and comparisons. *Behav Ecol Sociobiol* **65**, 23-35 (2011).
