# Supplementary material for: DNA sequence-dependent activity and base flipping mechanisms of DNMT1 regulate genome-wide DNA methylation
Source: Nat Commun. 2020 Jul 24;11:3723. doi: 10.1038/s41467-020-17531-8 (PMC7381644; doi:10.1038/s41467-020-17531-8)
Supplement: Supplementary file 3 — Reporting Summary [file 41467_2020_17531_MOESM3_ESM.pdf]

nature research

Corresponding author(s): Jeltsch

Last updated by author(s): 26.06.2020

Reporting Summary

Nature Research wishes to improve the reproducibility of the work that we publish. This form provides structure for consistency and transparency in reporting. For further information on Nature Research policies, see Authors & Referees and the Editorial Policy Checklist .

Statistics

For all statistical analyses, confirm that the following items are present in the figure legend, table legend, main text, or Methods section.

n/a	Confirmed	
☐	☒	The exact sample size (n) for each experimental group/condition, given as a discrete number and unit of measurement
☐	☒	A statement on whether measurements were taken from distinct samples or whether the same sample was measured repeatedly
☐	☒	The statistical test(s) used AND whether they are one- or two-sided<br>*Only common tests should be described solely by name; describe more complex techniques in the Methods section.*
☒	☐	A description of all covariates tested
☒	☐	A description of any assumptions or corrections, such as tests of normality and adjustment for multiple comparisons
☒	☐	A full description of the statistical parameters including central tendency (e.g. means) or other basic estimates (e.g. regression coefficient) AND variation (e.g. standard deviation) or associated estimates of uncertainty (e.g. confidence intervals)
☐	☒	For null hypothesis testing, the test statistic (e.g. F, t, r) with confidence intervals, effect sizes, degrees of freedom and P value noted<br>*Give P values as exact values whenever suitable.*
☒	☐	For Bayesian analysis, information on the choice of priors and Markov chain Monte Carlo settings
☒	☐	For hierarchical and complex designs, identification of the appropriate level for tests and full reporting of outcomes
☐	☒	Estimates of effect sizes (e.g. Cohen's d, Pearson's r), indicating how they were calculated

Our web collection on statistics for biologists contains articles on many of the points above.

Software and code

Policy information about availability of computer code

Data collection	X-ray diffraction data were collected from synchrotron beamline BL5.0.2 at Advanced Light Source (ALS), Lawrence Berkeley National Laboratory Laboratory and the 24-ID-C beamline at the Advanced Photon Source, Argonne National Laboratory using the standard software of the beamlines.
Data analysis	For structural study:<br>The HKL3000, PHENIX 1.15.2-3472-000, WinCoot 0.8.9 and Pymol v0.99 software were used for data processing and analysis.<br>For biochemical/epigenomic analysis:<br>Galaxy, Trim Galore!, Pear, Filter FASTQ, bwameth, BED tools, MS Excel Professional Plus 2016, MS Visual Basic 2010 were used for data analysis. All additional scripts used in this study for data analysis of the NGS kinetics are available upon request. The code for kinetic modelling is accessible at https://github.com/TheVincentWagner/SimModelDNAMethylation.git and directly executable with a python 3 distribution.

For manuscripts utilizing custom algorithms or software that are central to the research but not yet described in published literature, software must be made available to editors/reviewers. We strongly encourage code deposition in a community repository (e.g. GitHub). See the Nature Research guidelines for submitting code & software for further information.

Data

Policy information about availability of data

All manuscripts must include a data availability statement. This statement should provide the following information, where applicable:

- Accession codes, unique identifiers, or web links for publicly available datasets
- A list of figures that have associated raw data
- A description of any restrictions on data availability

Structural data have been submitted to PDB (codes 6W8W and 6W8V). DNMT1 NGS kinetic raw data have been uploaded at DaRUS, the Data Repository of the University of Stuttgart (DOI: 10.18419/darus-628 and DOI: 10.18419/darus-629. The code for kinetic modelling is accessible at https://github.com/

TheVincentWagner/SimModelDNAMethylation.git and directly executable with a python 3 distribution. All additional scripts used in this study for data analysis of the NGS kinetics are available upon request. Source data are provided with this paper.
NGS raw data refer to Fig. 1, 3, 6, and 7.
Structural raw data refer to Fig. 4, 5, S5, S6, and S7.
Source data are provided for Fig. 1, 3, 6, S2, S3, and S4.

Field-specific reporting

Please select the one below that is the best fit for your research. If you are not sure, read the appropriate sections before making your selection.

☒ Life sciences ☐ Behavioural & social sciences ☐ Ecological, evolutionary & environmental sciences

For a reference copy of the document with all sections, see nature.com/documents/nr-reporting-summary-flat.pdf

Life sciences study design

All studies must disclose on these points even when the disclosure is negative.

Sample size	N/A
Data exclusions	N/A
Replication	Biochemical experiments were conducted in independent duplicates or triplicates. The number of experiments is indicated.
Randomization	N/A
Blinding	N/A

Reporting for specific materials, systems and methods

We require information from authors about some types of materials, experimental systems and methods used in many studies. Here, indicate whether each material, system or method listed is relevant to your study. If you are not sure if a list item applies to your research, read the appropriate section before selecting a response.

Materials & experimental systems

n/a	Involved in the study	
☒	☐	Antibodies
☒	☐	Eukaryotic cell lines
☒	☐	Palaeontology
☒	☐	Animals and other organisms
☒	☐	Human research participants
☒	☐	Clinical data

Methods

n/a	Involved in the study	
☒	☐	ChIP-seq
☒	☐	Flow cytometry
☒	☐	MRI-based neuroimaging
