# Supplementary material for: DNA sequence-dependent activity and base flipping mechanisms of DNMT1 regulate genome-wide DNA methylation
Source: Nat Commun. 2020 Jul 24;11:3723. doi: 10.1038/s41467-020-17531-8 (PMC7381644; doi:10.1038/s41467-020-17531-8)
Supplement: Supplementary file 2 — Peer Review [file 41467_2020_17531_MOESM2_ESM.pdf]

Reviewers' comments:

Reviewer #1 (Remarks to the Author):

The authors analyzed and reported the specificity of DNMT1. They also solved the crystal structures of DNMT1 with favored or disfavored ds DNA, which explain why DNMT1 has specific enzymatic activity. This manuscript will be of interest to researchers working in the DNA methylation, DNA modification, epigenetic inheritance or protein-DNA interactions field.

Please go through the manuscript carefully, make sure no mistakes.

The "novel" should not be used in the paper.

Tables in the figures should be listed as table.

Please make Figure 1c and 3e using the same method, which will be easier to compare.

The +1 position is after "CG"? The Figure S1C should be modified.

PDB number in Table 2 is wrong for "4DA4"

The line 241 "GGCGGC" is different from what (AGGCGGGC) used in the table 2. I think the sequence in table 2 should be AGGCGGCC).

The table S4 should be put into the main text and put your PDB number after receiving your final PDB validation report.

In the line 251, it's better to say that "presumably due to the low affinity of the DNMT1 with this highly disfavored DNA". The authors can discuss this more.

The direction of dsDNA in the Figure S5c and S5d were labeled wrong, please be consistent with Figure 4f and 4g.

Please keep the orientation of Figure 4b exactly as in Figure 4a, as well as including protein background.

It is interesting to discuss the effect of disruption of hydrogen bond between G7 and G8' on the binding of GGCGGC to DNMT1.

Could the authors explain what kind of roles of the straight conformation play in CCG and ACG complexes, or (1) the catalytic helix of DNMT1 undergoes dynamics but not through straight conformation (2) just for the cofactor association and dissociation? If just for the association and dissociation of AdoHcy molecule (SAH), could you get Kd for SAH with these three different DNAs? In addition, the catalytic helix of DNMT1 with high rank DNA undergoes more dynamics (CCG compared to ACG), maybe authors can discuss this more.

In this regard, a subtle conformational change in the bound DNA (e.g.variation in the minor groove width) may influence the equilibrium between the two alternative conformations (Figure 5a-c).

How to influence? How to approve the straight conformation is existing in CCG or ACG complexes? If you can't approve this, it's better to say the catalytic helix of DNMT1 undergoes different dynamics with different DNA. It seems like the minor groove of GCG caused the formation of straight conformation, if DNA without that kind of minor groove, may not cause the formation of straight conformation.

Can authors get Kd for DNMT1 with these three ds DNAs by ITC, FP or other biophysical assays? It will be good to include this type of data.

Reviewer #2 (Remarks to the Author):

In this manuscript, the authors combined two pieces of data together: (1) the activity of full-length murine Dnmt1 on a long 349-bp naked DNA, aiming to find out the in vitro specificity for the neighboring sequence of CpG dinucleotide, and (2) two structures of C-terminal catalytic domain of Dnmt1 in complex with two short 12-bp oligonucleotides. I will comment on the relevance of the in vitro experimental design to mammalian maintenance DNA methylation and the quality of the X-ray structures. Based on the following assessment, the manuscript is suitable to a specialized Biochemistry journal.

1. Conceptual consideration. It was known that mammalian DNA methylation is established by de novo methylation (carried out by Dnmt3 family) in connection with the status of histone modifications (particularly H3K4 and K36). Maintenance methylation of hemimethylated CpG dinucleotides (carried out by Dnmt1) at replication forks is the key step of faithful mitotic inheritance of genomic methylation patterns, regardless of the sequence context of CpG dinucleotide. The work of epigenetic community in the last 10-20 years established that the DNA maintenance methylation, in addition to Dnmt1, required additional protein cofactor (UHRF1) and binding of specific histone modifications. A recent publication (PMID 32084344) discussed DNA maintenance methylation from an evolutionary perspective.

2. Experimental design consideration. Using a long 349-bp naked DNA containing 44 CpG sites (coupled with deep sequencing) is a step forward from the authors' own work published more than 10 years ago using short oligos containing single CpG. However, this experimental design neither reflects what happens during DNA replication nor considers other factors mentioned in point #1.

3. The authors compared flanking sequence specificities of Dnmt1, Dnmt3a and Dnmt3b (Figure 3B). Were the experiments done using the same set of DNA substrates? Do the three enzymes have the same or different specificities? If they are different, what would be the consequence for the maintenance enzyme to have different specificities from that of de novo enzymes? What will be the advantage or disadvantage for the evolutionary persistence of DNA methylation for millions of years?

4. The corresponding structural work uses the catalytic domain and a short oligo with a single CpG. Does the catalytic domain have the same selective specificity on the single-CpG containing oligo as that of full-length enzyme on the long oligo? Would the specificity be influenced by the allosteric activation of mouse Dnmt1 by the N-terminal part of the enzyme, as evident by the same authors' previous publication, but is not included in the structural study?

5. The quality of the X-ray structure is a significant concern. The electron density maps shown in Figure S5 is very poor and of low quality. Only half of the density is stable, and the other half is disordered. This discordance is very likely influenced by the crystal packing force, where one end of DNA molecule is in contact with a neighboring molecule, while the other loose end has no packing interaction. In order to fully understand the differences between base pairing seen in the structures, the authors will need to improve the structural quality of the DNA molecule probably by finding another crystallographic space group and/or using different constructs as well as different lengths of oligos (see point #4). Examples of base paring rearrangement and double flipping etc had been observed previously in bacterial DNA methyltransferases, SRA domains of UHRF1 from animal and plant and DNA repair enzymes. In each case, the observation was backed up by high quality structures, and will need to be cited. However, the current study does not have the same quality.

6. Abstract and text, “underlying molecular mechanism remains elusive” – authors need to be clear what remains elusive – the target specificity of CpG-flanking sequence of mouse Dnmt1 in an in vitro setting?

Page 7, “presumably due to the low stability of the DNMT1-DNA complex” – please explain.

The DNA sequence and length used for crystallization is different on p15 and on p20 (Table 2 legend).

Reviewer #3 (Remarks to the Author):

The authors analyze the enzymatic activity of DNMT1 by using in vitro hemi-methylated sequence, mathematical model, structural biology and genome-wide correlation analysis on WGBS/RRBS from the cell line. It is an important and interesting question to the epigenetic field. Most of the evidences are very solid. But there are still some major concerns:
1. For the last part of the “correlation of DNMT1 with genome-wide CpG methylation level”: to study the genome-wide activity of DNMT1, the best model is to use WGBS from DNMT single KO and wild type. Currently, there is already some publication about it, e.g. https://doi.org/10.1186/s13059-015-0685-2. Not sure why the authors use RRBS from 5-aza treated cell line. The 5-aza drug is nothing related with DNMT1.
2. From the genome-wide analysis part, it seems that the model works the best for the CpG island region (compare WGBS in Fig6a and RRBS in Fig6c/d). Since sequences in RRBS are mostly from CGI regions, if the author would like to claim this is a universal genome-wide model (which they claimed in the most part of the paper), they should exclude regions from CGI and CGI shore, and redo the correlation analysis again. Especially, recent studies on the gene-poor regions, such as PMD, suggest WCGW enriched for methylation loss during the replication (https://doi.org/10.1038/s41588-018-0073-4), which is different from the finding here. Also, recent findings on the hemi-methylated events at CTCF sites by using nascent DNA methylation may help the analysis here 10.1126/science.aan5480

Also, there are some minor concerns:
1. “CpG island upstream of the human SUHW1 gene”: Considering the direction of this gene in the genome, this CpG island should be downstream of SUHW1 (ZNF280A) (between ZNF280A and ZNF280B), unless the author used other CpG island annotation…
2. A459 cell: it might be the typo of A549?
3. The NGS data need to be deposit in the public database, such as GEO
4. The authors build libraries for 10bp fragment, which is hard for NGS. Also, the approach is the adapter ligation after bisulfite conversion, not sure if there is any bias here, since the adapter ligation after BS treatment usually brings some methylation bias. Also, for adapter trimming part in NGS from random 10mer, not sure paired-end or single-end is use, which platform in NGS is used. The details sometimes matter for adapter trimming. The author needs to show if the adapter sequence part is cleaned (fastqc result?)
5. For the first part of the result, the authors select a CGI from human genome but use murine DNMT1 for the reaction. Not sure the reason, but the best will be just select one CGI from the mouse.

General comment for all reviewers: We want to thank the three reviewers for their supportive and constructive comments. We appreciate the reviewers' view that "This manuscript will be of interest to researchers working in the DNA methylation, DNA modification, epigenetic inheritance or protein-DNA interactions field." (Reviewer 1), "It is an important and interesting question to the epigenetic field. Most of the evidences are very solid." (Reviewer 3), and "a step forward from the authors' own work published more than 10 years ago" (Reviewer 2). We have revised our manuscript to systematically address the major and minor concerns as outlined below. This is one of the instances where the suggested and executed experiments have very substantially improved the quality of the manuscript.

Reviewer #1

The authors analyzed and reported the specificity of DNMT1. They also solved the crystal structures of DNMT1 with favored or disfavored ds DNA, which explain why DNMT1 has specific enzymatic activity. This manuscript will be of interest to researchers working in the DNA methylation, DNA modification, epigenetic inheritance or protein-DNA interactions field.

Please go through the manuscript carefully, make sure no mistakes.

Reply: Thank you for this positive assessment. We have carefully read the manuscript once more and made all efforts to correct all remaining mistakes.

The "novel" should not be used in the paper.

Reply: The word "novel" has been removed.

Tables in the figures should be listed as table.

Reply: We thought about this but believe in Fig. 2 it will be very helpful to see the model parameters right next to the figures of the fit. In Fig. 3b and the new Fig. 7b, we use coloring of the table to increase readability. Based on these considerations, we like to propose to keep the figures as they are, but would be willing to change if our argumentation is not convincing.

Please make Figure 1c and 3e using the same method, which will be easier to compare.

Reply: Please note that different things are shown. In Fig. 1c, the sequence properties of the best and worst 20 sites among 44 sites in total are shown. Here the sample of 44 sites does not provide a full and equal coverage of all NNCGNN sites, hence a Weblogo plot is not appropriate. In Fig. 3e, 20 preferred and disfavored sites among all 256 NNCGNN sites are shown, which allows for Weblogo analysis and presentation. Most "test" readers of the manuscript preferred the Weblogo image over the observed/expected image, this is why we have shown it in Fig. 3e. The corresponding obs/exp plots are shown below. If requested we would be willing to change the images.

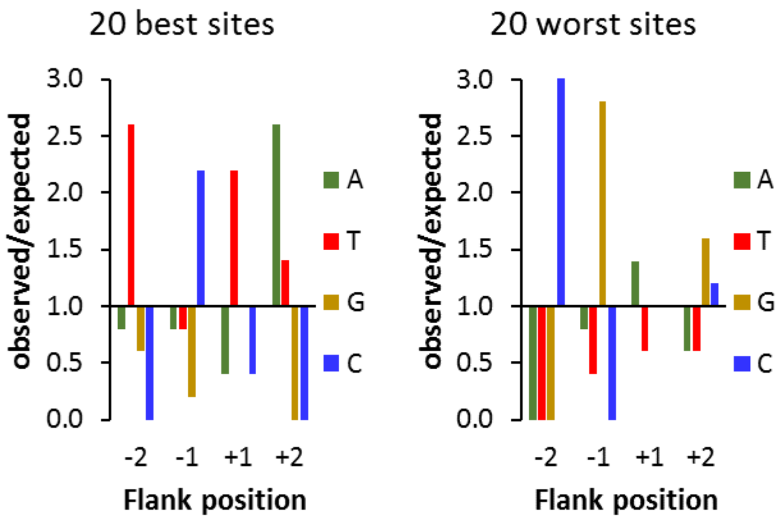

Figure R1: Observed/expected plots of the Weblogo data shown in Fig. 3e.

Furthermore, we have added one sentence in the main text, which explains that divergences between the random substrate methylation based flanking sequence profile and the results obtained with the 44-site substrate for the best sites are caused by a low representation of some bases at some flanking positions in the 44 CpG sites. This refers to A at -2, T at -1, T at +1, as well as A and T at +2 and explains all major differences between the enrichment/depletion profiles of both data sets. Similar as in the previous point, we certainly would be willing to change the data presentation if our argumentation is not convincing.

The +1 position is after “CG”? The Figure S1C should be modified.

Reply: We assume Fig. S1B is meant. Thank you, this mistake has been corrected.

PDB number in Table 2 is wrong for “4DA4”
The line 241 “GGCGGC” is different from what (AGGCGGGC) used in the table 2. I think the sequence in table 2 should be AGGCGGCC).

Reply: Both mistakes in Table 2 have been corrected.

The table S4 should be put into the main text and put your PDB number after receiving your final PDB validation report.

Reply: The table has been shifted as Table 3 into the main text, with the PDB numbers included.

In the line 251, it’s better to say that “presumably due to the low affinity of the DNMT1 with this highly disfavored DNA”. The authors can discuss this more.

Reply: We have changed the sentence to “presumably due to the low affinity of the DNMT1 for this sequence and/or low crosslinking yield with the 5fdC with this highly disfavored DNA sequence.” This sentence now also mentions the likely possibility that the crosslinking with 5fdC will be inefficient, because this depends on the first steps of the catalytic cycle.

The direction of dsDNA in the Figure S5c and S5d were labeled wrong, please be consistent with Figure 4f and 4g.

Reply: We have verified that the directions of dsDNA in Fig. S5c,d are correct and consistent with Fig. 4d (now Fig. 4b).

Please keep the orientation of Figure 4b exactly as in Figure 4a, as well as including protein background.

Reply: Thanks for pointing out this. Given the redundant presentation between Fig. 4b and 4d, we have removed the original Fig. 4b in the revised manuscript. Fig. 4b is oriented to fit to Suppl. Fig. 5.

It is interesting to discuss the effect of disruption of hydrogen bond between G7 and G8' on the binding of GGCGGC to DNMT1.

Reply: The reviewer's point is well taken. In the most disfavored complex a G is present at the -1 position (G8') where it forms a non-canonical base pair with the target site guanine (G7), which finally may trigger flipping of C7'. We have added a sentence to the discussion section highlighting the negative impact of a G at the -1 site. "The formation of this strong G7-G8' contact could explain the disfavor of G at the -1 site."

Could the authors explain what kind of roles of the straight conformation play in CCG and ACG complexes, or (1) the catalytic helix of DNMT1 undergoes dynamics but not through straight conformation (2) just for the cofactor association and dissociation? If just for the association and dissociation of AdoHcy molecule (SAH), could you get Kd for SAH with these three different DNAs? In addition, the catalytic helix of DNMT1 with high rank DNA undergoes more dynamics (CCG compared to ACG), maybe authors can discuss this more.

Reply: Following the reviewer's comment, we included the omit map for the two alternative conformations of the catalytic helix in the revised manuscript (Suppl. Fig. 7).
Given that the two mDNMT1-DNA complexes presented in this study and the mDNMT1-GCG complex reported earlier all capture the productive states of DNMT1, it is likely that the dynamic switch between the straight and kinked conformations serve to facilitate the turnover of the cofactor. On the other hand, we wish to clarify that the straight and kinked conformations coexist in all three complexes, despite with relatively different populations. Therefore, we consider that measurement of the Kd for SAH is not only technically challenging (production of covalent DNMT1-DNA complexes with sufficient yield), but also most likely it will not provide a clear-cut insight. Please note that our competitive methylation data of sequence libraries are not sensitive for all steps after methyl group transfer. Therefore, cofactor dissociation has not been discussed in our paper.

In this regard, a subtle conformational change in the bound DNA (e.g.variation in the minor groove width) may influence the equilibrium between the two alternative conformations (Figure 5a-c).

Reply: We agree and have added "e.g. variation in the minor groove width" to the sentence discussing the connection of DNA and protein conformations. "The equilibrium of these two alternative conformations appears to be influenced by the DNA conformation of the target strand (e.g. variation in the minor groove width) in particular in the segment downstream of the target C7' at flanking sites +1 and +2.".
Moreover, we have repeated the DNA shape analysis using an improved workflow, now allowing to analyse effects of minor groove width on all flanking positions (new Suppl. Fig. 8 replacing Suppl. Table 4). The data now show a clear and strong correlation of increased minor groove width at the plus-side of the CpG site with increased DNMT1 activity. This

result is in full agreement with the data reported in the previous version of the paper, which were restricted to the analysis of -1 to +1 flanks only.

How to influence? How to approve the straight conformation is existing in CCG or ACG complexes? If you can't approve this, it's better to say the catalytic helix of DNMT1 undergoes different dynamics with different DNA. It seems like the minor groove of GCG caused the formation of straight conformation, if DNA without that kind of minor groove, may not cause the formation of straight conformation.

Reply: We thank the reviewer for the comment. Inspection of the electron density maps for the ACG and CCG complexes also revealed the presence of straight conformation of the catalytic helix, albeit to a lower population. Following the reviewer's comment, we have provided the omit map for the two alternative conformations of the catalytic helixes in the revised manuscript.

Can authors get Kd for DNMT1 with these three ds DNAs by ITC, FP or other biophysical assays? It will be good to include this type of data.

Reply: We have conducted DNA binding gel shift experiments with the two different DNA molecules. As shown in Figure R2, there is no difference in the binding of the two substrates by full length DNMT1, which contrasts sharply with the different methylation rates observed for both substrates. This result indicates that the flanking sequence preferences mainly affect enzymatic turnover via transition state binding but not ground state binding. Similar results were obtained with mDNMT1(731-1602) (data not shown).

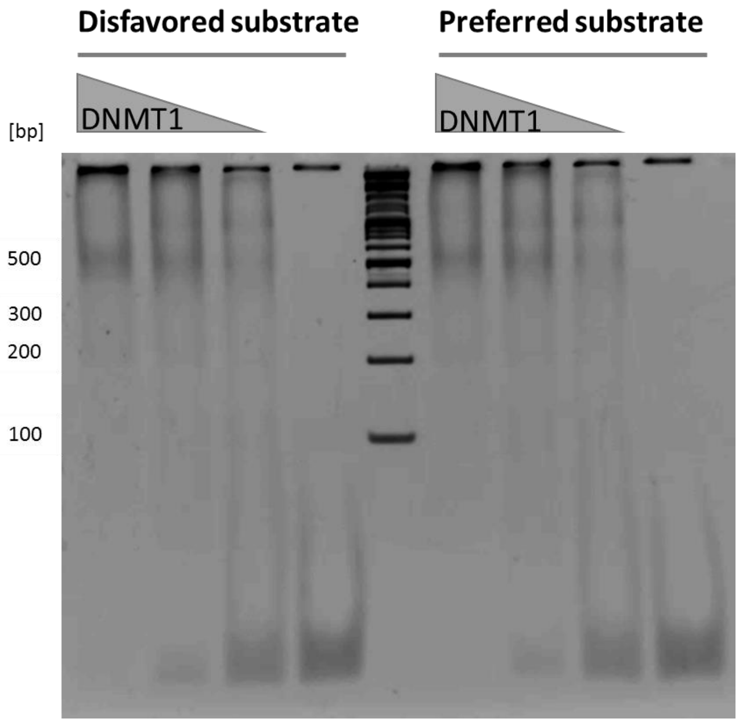

Figure R2: Gelshift of the designed disfavored or favored 30mer substrates with DNMT1. Method description: DNMT1 binding reactions were incubated for 30 min at 37°C after assembling the following mixture on ice: 0.122/0.244/0.488 µM DNMT1, 0.122 µM 30mer substrate, 1X methylation buffer (500 mM HEPES, 5 mM EDTA, 2.5 mM DTT, pH = 7.2 with KOH), containing 0.1 % Triton X-100, 0.5 mg/mL BSA, and 200 µM Sinefungin (Abcam). Different volumes of the DNMT1 protein were compensated by addition of dialysis buffer to keep the same salt and glycerol concentration. Afterwards, loading dye was added and samples were loaded on a 6% TB-Polyacrylamide gel and run for 75 min with 75 V and stained by GelRed.

Reviewer #2

In this manuscript, the authors combined two pieces of data together: (1) the activity of full-length murine Dnmt1 on a long 349-bp naked DNA, aiming to find out the in vitro specificity for the neighboring sequence of CpG dinucleotide, and (2) two structures of C-terminal catalytic domain of Dnmt1 in complex with two short 12-bp oligonucleotides. I will comment on the relevance of the in vitro experimental design to mammalian maintenance DNA methylation and the quality of the X-ray structures. Based on the following assessment, the manuscript is suitable to a specialized Biochemistry journal.

Reply: We understand the reviewer's concern, which regards two central questions of this study: (1) the conceptual advance of this study and (2) whether the structural studies are solid enough to support the central conclusion. As delineated below, we have addressed the reviewer's concern via new biochemical experiments, additional genome-wide methylation analysis as well as crystallographic analysis.

1. Conceptual consideration. It was known that mammalian DNA methylation is established by de novo methylation (carried out by Dnmt3 family) in connection with the status of histone modifications (particularly H3K4 and K36). Maintenance methylation of hemimethylated CpG dinucleotides (carried out by Dnmt1) at replication forks is the key step of faithful mitotic inheritance of genomic methylation patterns, regardless of the sequence context of CpG dinucleotide. The work of epigenetic community in the last 10-20 years established that the DNA maintenance methylation, in addition to Dnmt1, required additional protein cofactor (UHRF1) and binding of specific histone modifications. A recent publication (PMID 32084344) discussed DNA maintenance methylation from an evolutionary perspective.

Reply: The reviewer's point is well taken. As the reviewer pointed out, the role of DNMT1 in maintenance DNA methylation is supported by multiple regulatory mechanisms, including UHRF1-mediated targeting, histone modifications and intrinsic substrate preference for hemimethylated CpG sites. We have clarified this in the revised manuscript, and included a discussion of the evolutionary perspective raised by the recent paper. In fact, this has also been our research focus in previous studies.

We also wish to clarify that this study, through discovery of flanking sequence preferences of DNMT1, their mechanistic and structural origins and their effects on genome-wide DNA methylation patterns, further extends our view on the multi-layered regulation of DNMT1. Importantly, our work reveals flanking sequence-dependent base flipping mechanism of DNMT1, an observation that has not been reported for any DNMT previously. This observation provides novel insights into the mechanism of action of a key enzyme in the maintenance DNA methylation process. The high biological relevance of the DNMT1 flanking sequence effects is documented by the strong correlation of flanking sequence profiles of different published genome wide DNA methylation analysis with the flanking preferences of DNMT1 discovered here.

In the revised manuscript, we have also included additional bioinformatic analysis of methylation data from mouse ES cells (Li et al., 2015), which was triggered by reviewer 3. This analysis revealed a strong correlation of DNMT1 NNCGNN flanking sequence preferences with genome methylation patterns in wildtype mouse ES cells and in DKO cells containing a double deletion of DNMT3A and DNMT3B. In contrast, methylation patterns in DNMT1 knock-out ES cells (1KO) do not correlate with the DNMT1 sequence profile, but with profiles determined for DNMT3A and DNMT3B of our previous work. The very low

residual methylation signal in TKO cells (containing a triple knock-out of DNMT1, DNMT3A and DNMT3B) did not correlate with any of the DNMT preference profiles. This result clearly indicates that DNA methylation profiles in wildtype cells are heavily influenced by the flanking sequence preferences of DNMT1 – a conceptual advance that was not expected based on the existing literature.

2. Experimental design consideration. Using a long 349-bp naked DNA containing 44 CpG sites (coupled with deep sequencing) is a step forward from the authors' own work published more than 10 years ago using short oligos containing single CpG. However, this experimental design neither reflects what happens during DNA replication nor considers other factors mentioned in point #1.

Reply: We agree with the reviewer that DNMT1-mediated maintenance DNA methylation involves multiple factors in cells. We have included additional discussion on this in the revised manuscript. Meanwhile, we wish to clarify that this study focuses on the effect of the intrinsic enzymatic preference of DNMT1 on its substrate selectivity.

We also like to mention that in addition to the approach mentioned by the reviewer, our paper employs a novel kinetic analysis using hemimethylated substrate and we report novel structures of DNMTs, and novel flanking sequence dependent base flipping mechanisms. Therefore, the experiments conducted in this work go well beyond the stage established in previous papers by our groups and others.

3. The authors compared flanking sequence specificities of Dnmt1, Dnmt3a and Dnmt3b (Figure 3B). Were the experiments done using the same set of DNA substrates? Do the three enzymes have the same or different specificities? If they are different, what would be the consequence for the maintenance enzyme to have different specificities from that of de novo enzymes? What will be the advantage or disadvantage for the evolutionary persistence of DNA methylation for millions of years?

Reply: The substrates were the same and flanking sequence preferences are different indeed. This point has been clarified in the text now. This is clearly illustrated in the lack of correlation of preference profiles of DNMT1, DNMT3A and DNMT3B shown in Fig. 3b. Moreover, the novel analyses conducted with DNA methylation data from mouse ES cells with knock out of DNMTs now confirm that the flanking sequence preferences of the different enzymes directly influence genomic methylation profiles.
In addition, the reviewer raises the interesting and important question of the evolutionary connection of our findings. Therefore, we have added on paragraph discussing evolutionary implications of our findings at the end of the discussion.

In our view, there is no advantage value for a maintenance DNA methyltransferase in having flanking sequence preferences. So, we would not argue that they are an evolutionary adaptation. Instead, we believe that the necessity to form a tight and close DNA complex requires contacts of the DNMT to the DNA outside of the central CpG site. Even if no base specific contacts are formed this will result in flanking sequence preferences, because certain DNA conformations will be preferred over others. In this view, flanking sequence preferences would illustrate the limitations of the engineering capabilities of Nature not being able to set up an enzyme that is highly specific for hemimethylated CpG sites and ignores everything else on both sides. When following that view, the different preferences of DNMT1 and DNMT3A/3B are explainable, because both enzymes interact with the DNA in a different way. Hence, the overall deformations and forces in the complexes are different, explaining

different flanking sequence preferences. However, these considerations are rather speculative and we would prefer not to include them in the manuscript.

4. The corresponding structural work uses the catalytic domain and a short oligo with a single CpG. Does the catalytic domain have the same selective specificity on the single-CpG containing oligo as that of full-length enzyme on the long oligo? Would the specificity be influenced by the allosteric activation of mouse Dnmt1 by the N-terminal part of the enzyme, as evident by the same authors' previous publication, but is not included in the structural study?

Reply: Thank you for this suggestion. In the revised manuscript, we have also tested the flanking sequence preference of the truncated DNMT1(731-1602), which was used for the structural analysis. As shown in the new Suppl. Figure 4c, the obtained preferences reflect the results obtained with the full-length protein. As the reviewer pointed out, the allosteric regulation of Dnmt1 by the N-terminal domains influences its CpG specificity. However, it does not appear to influence its preference for the CpG-flanking sequences.

5. The quality of the X-ray structure is a significant concern. The electron density maps shown in Figure S5 is very poor and of low quality. Only half of the density is stable, and the other half is disordered. This discordance is very likely influenced by the crystal packing force, where one end of DNA molecule is in contact with a neighboring molecule, while the other loose end has no packing interaction. In order to fully understand the differences between base pairing seen in the structures, the authors will need to improve the structural quality of the DNA molecule probably by finding another crystallographic space group and/or using different constructs as well as different lengths of oligos (see point #4).

Reply: The reviewer's point is well taken. The crystallization condition used for the mDNMT1-DNA complexes was the only condition identified through extensive screening and choices of various constructs, as reported earlier. We wish to clarify that the two mDNMT1-DNA complexes presented in this study share nearly identical crystal packing with our previously determined structure of mDNMT1-CCG complex (2.6 Å resolution) (Figure R3, A-C), which rules out that the observed difference in dynamics is due to crystal packing conditions. As pointed out by the reviewer, the three complexes share a similar quality of electron density at region 3' to the methylation sites (fC7'), but different quality of electron density on the 5' flanking regions (Figure R3, D-I). In fact, the region with relatively poor electron density coincides with the base pair rearrangement (Figure R3, D-I). Therefore, we attribute the disorder of this region to its intrinsic dynamics, which, as the reviewer may well know, is a factor that often substantially influences the quality of local electron density map (Figure R3). To clarify the structural quality of the DNA model in each of the complexes, we have generated new Fo-Fc omit map of these DNAs contoured at 1.3 σ level. These new maps indicate that our structural models are highly reliable.

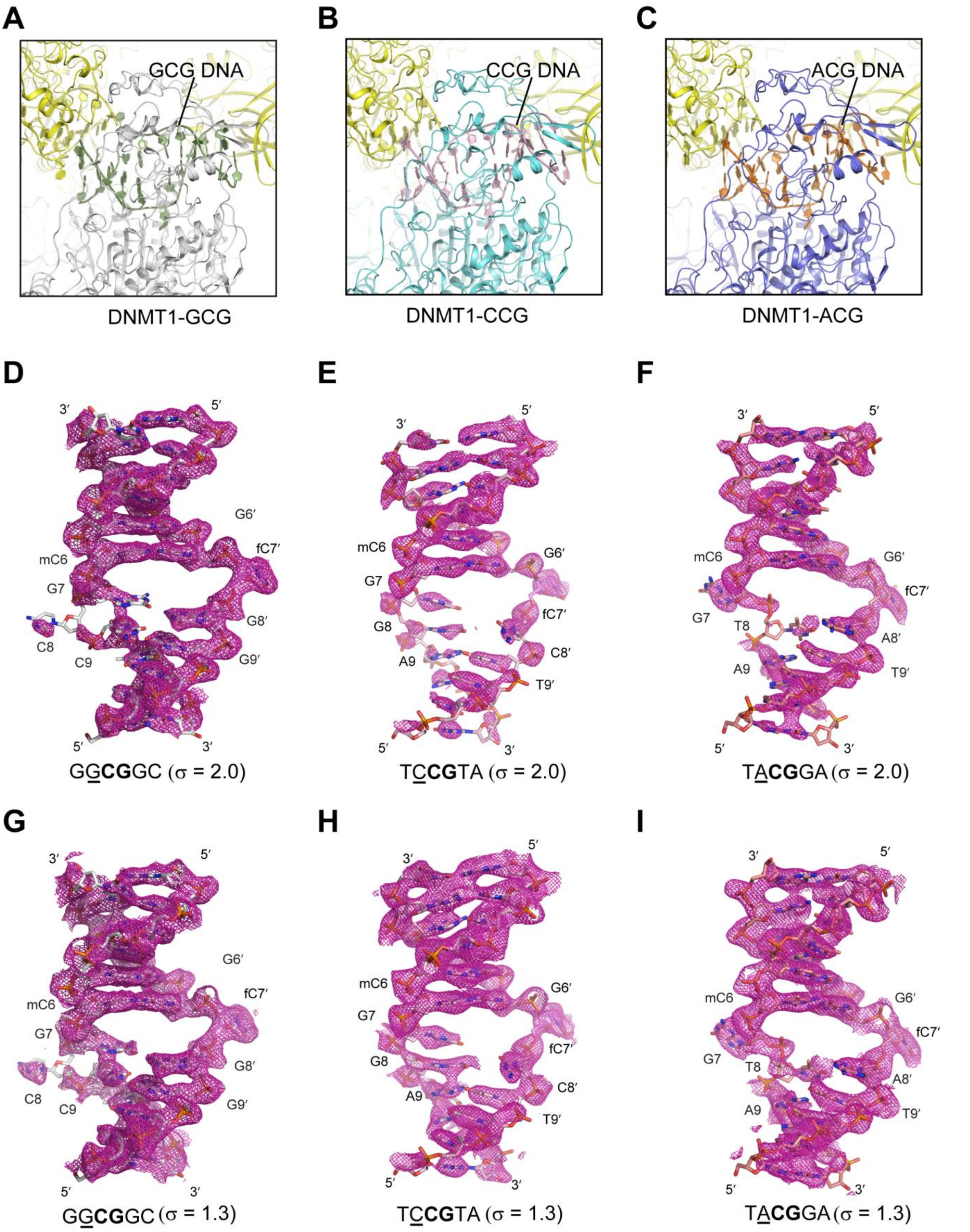

Figure R3. (A-C) Crystal packing interaction of the mDNMT1-GCG (A), mDNMT1-CCG (B) and mDNMT1 DNMT1-ACG (C) complexes. Note that three complexes engage a similar crystal packing interaction with the symmetry-related mDNMT1 complex (yellow). (D-F) Fo-Fc omit map of the DNA in the mDNMT1-GCG (D), mDNMT1-CCG (E) and mDNMT1-ACG (F) complexes, contoured at 2.0 σ level. (G-I) Fo-Fc omit map of the DNA in the DNMT1-GCG (G), DNMT1-CCG (H) and DNMT1-ACG (I) complexes, contoured at 1.3 σ level.

Examples of base paring rearrangement and double flipping etc had been observed previously in bacterial DNA methyltransferases, SRA domains of UHRF1 from animal and plant and DNA repair enzymes. In each case, the observation was backed up by high quality structures, and will need to be cited. However, the current study does not have the same quality.

Reply: We agree with the reviewer on the literature of base flipping. We also wish to clarify that the dual base flipping mechanism associated with the mDNMT1-DNA complex has already been reported in our earlier study. However, in the present study, we observed DNA sequence context-dependent base flipping mechanisms of DNMT1. Such an observation has not been made for any other protein so far, and in our view this greatly extends our understanding of the structure and mechanism of DNMT1-mediated DNA methylation and of base flipping in general. We thank the reviewer for pointing out that base paring rearrangement and double flipping have previously been observed for many other proteins. As the reviewer may well know, base flipping triggered by most of these proteins involve the insertion of flipped base into a well-defined binding pocket, rather than extrusion into solvent as observed for DNMT1. Following the reviewer's comments, we have included a discussion on the base flipping by other methyltransferases in the revised manuscript as follow.
"These alternative base flipping mechanisms of DNMT1 add a new example to the large family of DNA interacting proteins involving base flipping (Cheng & Roberts, 1998} and may provide clues for other systems. For example, it has previously been observed that prokaryotic cytosine-C5 methyltransferases like M.HhaI (GCGC) (Klimasauskas, et al. 1994) and M.HaeIII (GGCC) (Reinisch, et al. 1995) follow different base flipping and rearrangement mechanisms. Our data highlight the general role of the different DNA sequences surrounding the target cytosine in these enzymes, which can be different target sequences as in M.HhaI and M.HaeIII or different flanking sequences surrounding a CpG sites as in DNMT1."

6. Abstract and text, "underlying molecular mechanism remains elusive" – authors need to be clear what remains elusive – the target specificity of CpG-flanking sequence of mouse Dnmt1 in an in vitro setting?

Reply: The abstract has been rephrased to "but the molecular mechanisms connecting DNA methylation patterns and enzyme activity remain elusive" In the introduction, the corresponding sentence has been changed to: "Due to lack of systematic structural and kinetic characterizations, the mechanistic principles underlying the target specificity of DNMT1 and its connection with genomic methylation patterns remain elusive."

Page 7, "presumably due to the low stability of the DNMT1-DNA complex" – please explain.

Reply: We have attempted to purify the mDNMT1- CGCGAG DNA complex under the same condition as used for other mDNMT1-DNA complexes, but the yield has proved to be extremely low. We have changed the sentence to "presumably due to the low affinity of the DNMT1 for this sequence or low crosslinking yield with the 5fdC with this highly disfavored DNA sequence." This sentence now also mentions the likely possibility that the crosslinking with 5fdC will be inefficient, because this depends on the first steps of the catalytic cycle.

The DNA sequence and length used for crystallization is different on p15 and on p20 (Table 2 legend).

Reply: The corresponding sentence in the Table 2 legend has been removed.

Reviewer #3

The authors analyze the enzymatic activity of DNMT1 by using in vitro hemi-methylated sequence, mathematical model, structural biology and genome-wide correlation analysis on WGBS/RRBS from the cell line. It is an important and interesting question to the epigenetic field. Most of the evidences are very solid. But there are still some major concerns:

1. For the last part of the "correlation of DNMT1 with genome-wide CpG methylation level": to study the genome-wide activity of DNMT1, the best model is to use WGBS from DNMT single KO and wild type. Currently, there is already some publication about it, e.g. https://doi.org/10.1186/s13059-015-0685-2. Not sure why the authors use RRBS from 5-aza treated cell line. The 5-aza drug is nothing related with DNMT1.

Reply: The reviewer's point is well taken. The appropriateness of the 5-azacytidine system is given by two considerations: First, 5-azacytidine is clinically relevant. Hence it is of interest to understand its mechanism of action in more details. Second, the demethylation effect of 5-azacytidine is caused by the deactivation of active DNA methyltransferases in cells, leading to DNA replication under conditions of insufficient DNMT activity finally resulting in gradual passive DNA demethylation. As DNMT1 is the main DNA methyltransferase in differentiated cells and it carries the highest load in maintenance methylation, the effect of 5-aza is mainly due to the inhibition of DNMT1.

We appreciate the suggestion to analyze the mouse methylation data as well. This analysis has been carried out as proposed and the results are now presented in our manuscript. They strongly support our conclusions and very clearly document the important biological effect of flanking sequence preferences of DNMTs (including DNMT1) in the generation of biological DNA methylation patterns. Strikingly, the analysis of NNCGNN genome methylation profiles in wildtype mouse ES cells, DNMT1 knock-out ES cells (1KO), DNMT3A/DNMT3B double knock-out ES cells (DKO) and DNMT1/DNMT3A/DNMT3B triple knock-out ES cells (TKO) (Li et al., 2015) revealed a very strong correlation of wt ES cell methylation patterns with DNMT1 flanking sequence preferences. This correlation was lost in 1KO and TKO cells, but even stronger in DKO cells. Next, we also tested the correlation of these methylation profiles with sequence preferences determined for DNMT3A and DNMT3B in our previous work (referring to Fig. 3b of the DNMT1 manuscript) and also an average profile of DNMT3A and DNMT3B. Strikingly, while low correlation of wildtype, DKO and TKO methylation patterns were observed with DNMT3 profiles, the genomic methylation of 1KO cells was highly correlated with the DNMT3 flanking sequence preferences. This result clearly indicates that DNA methylation profiles in wildtype cells are heavily influenced by the flanking sequence preferences of DNMT1.

2. From the genome-wide analysis part, it seems that the model works the best for the CpG island region (compare WGBS in Fig6a and RRBS in Fig6c/d). Since sequences in RRBS are mostly from CGI regions, if the author would like to claim this is a universal genome-wide model (which they claimed in the most part of the paper), they should exclude regions from CGI and CGI shore, and redo the correlation analysis again. Especially, recent studies on the gene-poor regions, such as PMD, suggest WCGW enriched for methylation loss during the replication (https://doi.org/10.1038/s41588-018-0073-4), which is different from the finding here. Also, recent findings on the hemi-methylated events at CTCF sites by using nascent DNA methylation may help the analysis here 10.1126/science.aan5480

Reply: We have repeated the GSM182522 data set analysis after splitting the data into CGIs,

CGI shores, and the rest of the genome (Table R1). The data indicate that all genome compartments contribute to the overall correlation of DNMT1 activity with genomic methylation patterns, because correlation factors are highest if all data are included.

Table R1: Pearson correlation factors of genomic methylation of NNCGNN flanks within the GSM182522 data set. In the first line of the table, the overall methylation patterns are compared with results of analyses restricted on CGIs, CGI shores and the rest of the genome. In the second line the correlation factors of the different genome methylation patterns with the DNMT1 activity pattern are shown.

	All	CGI	Shore	Rest
**Correlation with overall genome methlyation**	1.00	0.56	0.79	0.72
**Correlation with DNMT1**	0.41	0.13	0.22	0.31

The Zhou et al. paper mentioned by the reviewer identified a preference of WCGW sites to become demethylated in a very large meta-dataset. This effect does not match to our DNMT1 preference profiles, suggesting that other factors, like a preferred occupancy of these sites by transcription factors or the enrichment of sites in repeats that are prone to demethylation are responsible for this effect. We have included additional discussion on this in the revised manuscript.

Also, there are some minor concerns:
1. "CpG island upstream of the human SUHW1 gene": Considering the direction of this gene in the genome, this CpG island should be downstream of SUHW1 (ZNF280A) (between ZNF280A and ZNF280B), unless the author used other CpG island annotation…

Reply: There was a mistake in the gene name. The CGI is upstream of the SUHW2 gene, which has the current name ZNF280B. We have corrected this mistake und updated the name. We like to apologize for the confusion caused.

2. A459 cell: it might be the typo of A549?

Reply: This error has been corrected.

3. The NGS data need to be deposit in the public database, such as GEO

Reply: The data have been deposited in a public research data repository housed by Stuttgart University. The data are not genome based, so we believe GEO is not the most appropriate place.

4. The authors build libraries for 10bp fragment, which is hard for NGS. Also, the approach is the adapter ligation after bisulfite conversion, not sure if there is any bias here, since the adapter ligation after BS treatment usually brings some methylation bias. Also, for adapter trimming part in NGS from random 10mer, not sure paired-end or single-end is use, which platform in NGS is used. The details sometimes matter for adapter trimming. The author needs to show if the adapter sequence part is cleaned (fastqc result?)

Reply: The fragments used for library generation have a length of 67 bps, the sequenced insert is 122 bps. This is schematically shown in Suppl. Fig. 1b and has been clarified in the method description now. Based on this, it can be excluded that the adapter ligation will be influenced

by the methylation level of a single CpG site in the center of the fragment. Trimming of sequences can be conducted using standard procedures.

5. For the first part of the result, the authors select a CGI from human genome but use murine DNMT1 for the reaction. Not sure the reason, but the best will be just select one CGI from the mouse.

Reply: Murine and human CGIs are not differing in key sequence based features as illustrated below (Figure R4). The human CGI was just taken as an example CGI substrate with "typical" properties.

Figure R4: Compilation of key sequence based properties of mouse and human CGIs.

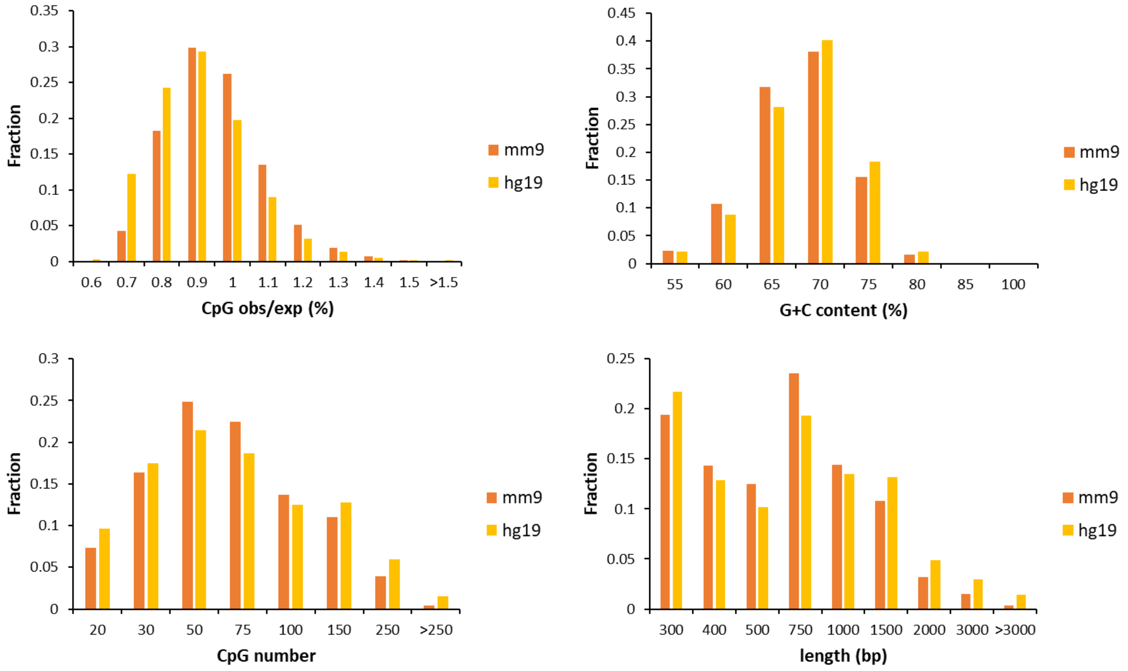

REVIEWERS' COMMENTS:

Reviewer #1 (Remarks to the Author):

Minor comments

Line 274, 302
Should figure 4c be replaced by Figure 4a?

Reviewer #2 (Remarks to the Author):

After reading the manuscript and the authors' response, I concluded that the revised manuscript didn't address my main concerns. Actually, based on the authors' response, it raised more troubling questions.

Significance - In response to my previous questions, the authors stated, on p6 of the rebuttal, that for the de novo enzymes Dnmt3a and Dnmt3b and the maintenance enzyme Dnmt1, "the substrates were the same and flanking sequence preferences are different indeed" and "In our view, there is no advantage value for a maintenance DNA methyltransferase in having flanking sequence preferences". This conclusion conflicts directly with the significance of the study regarding the in vitro "optimized" DNA substrates and Dnmt1-medaited CpG-flanking sequences (as stated in the Abstract). The overwhelming data in the literature demonstrated that the function of Dnmt1, together with a sophisticated regulatory machinery, is for faithful inheritance of genomic methylation patterns (hence the meaning of maintenance), but not to create its own sequence preferences (which the authors concluded there is no advantage value).

Molecular mechanism - Another puzzling statement on p7 - the allosteric regulation of Dnmt1 influences its CpG specificity but does not appear to influence its preference for the CpG-flanking sequences - discredits the outcome of the study on Dnmt1's intrinsic preference on naked long DNA. How does the catalytic domain of Dnmt1 achieve this selective regulation on the same piece of DNA? How does the conclusion statement contribute to the "the molecular mechanisms connecting DNA methylation patterns and enzyme activity"?

Structural quality - The concern on the quality of the X-ray structures is not adequately addressed. The authors provided Fo-Fc omit maps at lower sigma level (1.3 sigma) (p8) (!). This is the opposite of what conventional crystallographers will do. There are two common types of electron density maps used by researchers, the 2Fo-Fc map and Fo-Fc map – The former is typically shown in 1-2 sigma level, the latter is shown in higher sigma level (4-5 sigma or above). The authors can omit the target Cyt, and calculate the Fo-Fc omit map and see at which (highest) sigma level the base density would show up. Using this sigma level as an internal control, could the authors include the Fo-Fc omit maps for other parts of DNA in the manuscript?

Crystallographic contacts - Based on the information provided by the authors, the crystal contains two protein-DNA complexes (A and B), with one end of DNA molecule of complex A stacking against the corresponding end of complex B (head-to-head). It just happens that the end involved in the stacking interaction has more stable density and the loose end is more disordered. The authors did not distinguish whether the difference is due to the crystal packing interaction (between the two complexes within the asymmetric crystallographic unit) or the intrinsic dynamics of DNA sequence. The latter could be studied by different approaches, but the static nature of crystallography using a short piece of DNA with one loose end in this case is insufficient to explain the observation derived from a long piece of DNA used in the enzymatic assay.

Reviewer #3 (Remarks to the Author):

The authors have already addressed my concerns.

REVIEWERS' COMMENTS

Reviewer #1 (Remarks to the Author):
Minor comments
Line 274, 302
Should figure 4c be replaced by Figure 4a?
Reply: This has been corrected. Thank you.

Reviewer #2 (Remarks to the Author):
After reading the manuscript and the authors' response, I concluded that the revised manuscript didn't address my main concerns. Actually, based on the authors' response, it raised more troubling questions.
Significance - In response to my previous questions, the authors stated, on p6 of the rebuttal, that for the de novo enzymes Dnmt3a and Dnmt3b and the maintenance enzyme Dnmt1, "the substrates were the same and flanking sequence preferences are different indeed" and "In our view, there is no advantage value for a maintenance DNA methyltransferase in having flanking sequence preferences". This conclusion conflicts directly with the significance of the study regarding the in vitro "optimized" DNA substrates and Dnmt1-medaited CpG-flanking sequences (as stated in the Abstract). The overwhelming data in the literature demonstrated that the function of Dnmt1, together with a sophisticated regulatory machinery, is for faithful inheritance of genomic methylation patterns (hence the meaning of maintenance), but not to create its own sequence preferences (which the authors concluded there is no advantage value).
Reply: Please note that, as shown in Fig. 6a and b of our manuscript, the flanking sequence preferences of DNMT1 modulate genomic methylation levels mainly between 60 and 90% methylation, but they do not determine, if a site is methylated or not methylated at all. This point has been further clarified in the results description and discussion of our manuscript. Wording in the abstract has been changed from "optimized" to "preferred".

Molecular mechanism - Another puzzling statement on p7 - the allosteric regulation of Dnmt1 influences its CpG specificity but does not appear to influence its preference for the CpG-flanking sequences - discredits the outcome of the study on Dnmt1's intrinsic preference on naked long DNA. How does the catalytic domain of Dnmt1 achieve this selective regulation on the same piece of DNA? How does the conclusion statement contribute to the "the molecular mechanisms connecting DNA methylation patterns and enzyme activity"?
Reply: Please consider that allosteric regulation of DNMT1 via the RFTD and CXXC domains controls the accessibility of the active site. This affects all potential target substrate DNAs irrespective of the flanking sequence of the CpG site. If the active site is accessible, it forms close contacts to the bound substrate DNA and substrates with different flanking sequences show distinct interactions with the active site leading to flanking sequence preferences. Moreover, distinct and flanking sequence dependent base flipping mechanisms can take only effect in the catalytically active complex, which triggers base

flipping. However, in the context of the specificity of DNMT1 for hemimethylated DNA, the flanking sequence preferences of the active site could act together with putative flanking sequence preferences of the CXXC domain. This point has been added to the discussion of our manuscript.

Structural quality - The concern on the quality of the X-ray structures is not adequately addressed. The authors provided Fo-Fc omit maps at lower sigma level (1.3 sigma) (p8) (!). This is the opposite of what conventional crystallographers will do. There are two common types of electron density maps used by researchers, the 2Fo-Fc map and Fo-Fc map – The former is typically shown in 1-2 sigma level, the latter is shown in higher sigma level (4-5 sigma or above). The authors can omit the target Cyt, and calculate the Fo-Fc omit map and see at which (highest) sigma level the base density would show up. Using this sigma level as an internal control, could the authors include the Fo-Fc omit maps for other parts of DNA in the manuscript?
Reply: We have chosen to contour the Fo-Fc omit map at sigma level 1.3 in order to validate our structural model. At higher contour level, it is apparent that the electron density of the -1 bases of CCG and ACG complexes fades away much faster than that of the target Cyt, presumably due to the intrinsic flexibility of the -1 sites.

Crystallographic contacts - Based on the information provided by the authors, the crystal contains two protein-DNA complexes (A and B), with one end of DNA molecule of complex A stacking against the corresponding end of complex B (head-to-head). It just happens that the end involved in the stacking interaction has more stable density and the loose end is more disordered. The authors did not distinguish whether the difference is due to the crystal packing interaction (between the two complexes within the asymmetric crystallographic unit) or the intrinsic dynamics of DNA sequence. The latter could be studied by different approaches, but the static nature of crystallography using a short piece of DNA with one loose end in this case is insufficient to explain the observation derived from a long piece of DNA used in the enzymatic assay.
Reply: We wish to clarify that all three DNMT1-DNA complexes (GCG, CCG and ACG) involve a similar crystal packing mode, as illustrated in our previous rebuttal letter. Therefore, their distinct base flipping behaviors are unlikely attributed to the crystal packing effects. Moreover, please note that the congruence of results obtained by crystallography and by biochemical experiments in solution strongly argues against a crystallographic artefact.

Reviewer #3 (Remarks to the Author):
The authors have already addressed my concerns.
Reply: Thank you.
